# Pre-plaque glutamatergic hyperexcitability, mitochondrial dysfunction, and dendritic remodeling in the hippocampus of one-month-old 5xFAD mice

**DOI:** 10.3389/fnagi.2026.1804332

**Published:** 2026-07-15

**Authors:** Amanda R. Kelley, Emily Sackinger, Mathew Frischman, Nicholas Thomas, Kaitlyn Kim, Grace Scuderi, Edwin M. Labut, Duncan MacMurchy, Toren Ikea-Mario, Jacob Rauenhorst, Easton Neitzel, Tacita Vu, Leda Liko, Alexandra Hoff, Ashley Hoff, Olivia Wallace, Wren K. E. Harry, Benjamin Hagen, Alejandro Z. Bihun, Ibrahim A. Abou-Seada, Ken Lee, Judy Butler, Fikru Nigussie, Arpa Ebrahimi, Phoebe Y. Lee, Luke C. Marney, Claudia S. Maier, Kathy R. Magnusson, Tory M. Hagen

**Affiliations:** 1 Linus Pauling Institute, Oregon State University, Corvallis, OR, United States; 2 Department of Biochemistry and Biophysics, Oregon State University, Corvallis, OR, United States; 3 Department of Biomedical Sciences, Carlson College of Veterinary Medicine, Oregon State University, Corvallis, OR, United States; 4 Department of Environmental and Molecular Toxicology, Oregon State University, Corvallis, OR, United States; 5 Department of Chemistry, College of Science, Oregon State University, Corvallis, OR, United States

**Keywords:** 5xFAD, amyloid-β (Aβ), hippocampus CA1, mitochondrial dysfunction, neuronal hyperexcitability

## Abstract

Alzheimer’s disease (AD) is characterized by progressive cognitive decline and stereotyped neuropathology, yet the earliest cellular events that precede overt plaque burden and measurable behavioral impairment remain incompletely defined. Here, we tested the hypothesis that synaptic hyperexcitability and subcellular metabolic dysfunction emerge early in the 5xFAD mouse model and contribute to region-specific neuronal vulnerability before substantial amyloid plaque deposition. Using the 5xFAD heterozygous mouse, we first established the onset of transgene expression and the timing of plaque accumulation. Robust transgene expression was detected by postnatal day 15 and statistically significant plaque accumulation in the CA1 stratum radiatum by 4 months of age. Hippocampal slice electrophysiology revealed an early hyperexcitable phenotype at 1 month of age, including both increased AMPA receptor-mediated transmission and N-methyl-D-aspartate receptor signaling associated with the GluN2B subunit. Given the tight coupling between glutamatergic hyperactivity, oxidative stress, calcium dysregulation, and mitochondrial health, we assessed mitochondrial structure and function at this pre-plaque stage. Mitochondrial abnormalities consistent with impaired bioenergetic homeostasis were evident within hippocampal synaptic processes. Morphological analyses demonstrated that these early changes were associated with altered dendritic architecture in the CA1 and dentate gyrus regions, revealing hippocampal subregional susceptibility. Finally, spatial transcriptomics identified regionally enriched molecular signatures consistent with differential vulnerability. The CA1 subregion exhibited pronounced downregulation of mitochondria-related transcripts, and single-cell deconvolution resolved this transcriptomic suppression specifically to CA1 pyramidal neurons (CA1.ProS); CA3 and dentate gyrus did not show equivalent mitochondrial pathway suppression. Together, these findings define a pre-plaque window in 5xFAD mice marked by GluN2B-linked glutamatergic hyperexcitability, early mitochondrial disruption, and selective dendritic and transcriptional vulnerability. Mitochondrial transcriptomic suppression was anatomically restricted to CA1 pyramidal neurons, establishing a cell-type-specific bioenergetic signature at 1 month of age, well before overt amyloid pathology. While the observations herein are descriptive in nature and detailed mechanisms have yet to be established, nevertheless, the integrated timeline suggests that synaptic and metabolic dysfunctions arise before substantial plaque deposition and may represent tractable early targets for intervention in AD.

## 1 Introduction

Alzheimer’s disease (AD) is a progressive neurodegenerative disorder that increasingly impairs memory and cognition, ultimately robbing the patient of the ability to carry out tasks of daily living (National Institute on Aging, 2016). The disease represents a major and growing public health burden: in the United States, an estimated 6.9 million individuals are currently living with AD, with total payments costing $360 billion and 13.8 million Americans are projected to be living with the disease by 2060 (Alzheimer’s Association, 2024 #2197). There is no cure for AD, and current treatments are mainly palliative in nature (Buccellato et al., 2023; Sheikh et al., 2023).

There appears to be no single cause for the sporadic forms of AD; rather, a complex interplay of environmental, biological, and genetic factors are involved to varying degrees (Zhang et al., 2021; Mertas and Bosgelmez, 2025). Because predispositional factors may occur decades prior to AD onset (Jack et al., 2013; Selkoe and Hardy, 2016), definitive early hallmarks for preventative strategies have been daunting to discover, and chemopreventive strategies have been difficult if not impossible to implement.

At the neuro-anatomical level, neurite dystrophy, loss of dendritic complexity and spine loss in pyramidal neurons of the CA1 hippocampus and cortex are consistently evident in post-mortem brains of AD patients (Perez-Cruz et al., 2011; Shi et al., 2020). Often this dystrophy is evident close to amyloid-β (Aβ) plaques (Blazquez-Llorca et al., 2017; Grutzendler et al., 2007). The temporal relationship between plaque formation and these anatomical changes is nuanced and model-dependent. In some rodent models, synaptic transmission impairment precedes plaque deposition (Hermann et al., 2009) and synapse loss mediated by complement and microglia activation also occurs earlier (Hong et al., 2016). However, in other studies, amyloid plaque formation precedes dendritic spine loss (Bittner et al., 2012). Hippocampal hyperexcitability also can emerge well before significant plaque burden (Ray et al., 2024; Busche et al., 2012). The temporal relationship is therefore model- and measure-dependent, making systematic pre-plaque characterization of specific models a valuable scientific goal.

The signature pathologies of AD are amyloid plaques and neurofibrillary tangles (Mirra et al., 1991; Armstrong, 2006). Familial and early onset AD is strongly associated with mutations in presenilin (PSEN) and amyloid precursor protein (APP) genes (Wu et al., 2012). PSEN is a subunit of gamma-secretase, which processes APP to Aβs of 38–43 amino acids (Esler and Wolfe, 2001; Takami et al., 2009). When transgenically expressed in AD mouse models, mutations in PSEN and APP tend to produce more Aβ42 than in non-transgenic mice (Citron et al., 1998; Eckman et al., 1997). Evidence suggests that amyloid accumulation begins up to 3 decades before dementia onset in humans (Masters et al., 2015; Bateman et al., 2012), highlighting the importance of targeting early disease events.

Based on the hallmark pathologies of AD, numerous transgenic animal models have been developed to replicate amyloidosis and/or tau pathology. The most widely used amyloid-overexpression models exhibit distinct phenotypic timelines. The PDAPP (J20; contains human mutant APP) mouse develops plaques by 6–10 months with hippocampus-dependent memory deficits by 12 months, but lacks tau pathology (Karl et al., 2012; Mucke et al., 2000). The APPswe/PSEN1ΔE9 mouse develops plaques beginning at 6–8 months with cognitive deficits by 18 months (Savonenko et al., 2005; Jankowsky et al., 2004). The 3xTg-AD mouse carries APP, PSEN1, and tau transgenes and develops plaques at 6 months and memory retention deficits at 4 months (Billings et al., 2005; Oddo et al., 2003). The 5xFAD mouse, the model that expresses the 5 most common FAD mutations in PSEN and APP, shows earlier plaque appearance, by 2–4 months, and memory problems by 3–6 months (Jawhar et al., 2012; Oakley et al., 2006). Among highly utilized early onset AD models, comparative transcriptomic analysis shows that the 5xFAD mouse exhibits the strongest positive correlation with late-onset human AD gene expression profiles (Preuss et al., 2020). Additionally, the 5xFAD model’s aggressive amyloid production timeline permits pre-plaque pathology investigation within the first postnatal month. Characterizing what changes arise in this earliest window could provide insights for both familial and sporadic AD and was the primary goal of the present study.

Multiple strains of PSEN mutant mice show a transient increase in N-methyl-D-aspartate receptor (NMDAR) transmission at an early age (3 months), followed by deficits later in life (Auffret et al., 2009, 2010; Dewachter et al., 2008; Wang et al., 2009). This has been shown to be associated with an increase in NMDA receptor GluN2B subunit expression in the PS1A246E PSEN mutant (Dewachter et al., 2009). Glutamate receptors (NMDARs and AMPARs) are found in high density in AD-affected brain regions such as hippocampus and cortex (Magnusson, 1998; Magnusson and Cotman, 1993). While NMDARs normally support memory and synaptic plasticity (Morris et al., 1986), overstimulation leads to calcium overload-related excitotoxicity (Choi, 1992). NMDARs conduct Ca^2+^ and there is an abnormal rise in internal Ca^2+^ in PSEN mutant mice (Schneider et al., 2001), which could potentially involve Aβ and GluN2B subunits (Ferreira et al., 2015). This rise was attributed to alterations in storage of calcium in the endoplasmic reticulum (Schneider et al., 2001), however, it could also be due to mitochondrial dysfunction (Stanika et al., 2009; Ma et al., 2020; Ferreira et al., 2015; Ryan et al., 2020; Mustaly-Kalimi et al., 2025; Walters and Usachev, 2023). Mitochondria serve as critical synaptic calcium buffers, and impaired mitochondrial calcium uptake can amplify NMDAR-driven calcium overload in a feed-forward manner. Investigation of the 5xFAD model – which carries two PSEN1 mutations alongside three APP mutations and develops amyloid pathology – is therefore a logical next step for determining whether NMDAR hyperactivity, calcium dysregulation, and mitochondrial injury co-occur in the context of amyloid-driven neurodegeneration.

5XFAD transgenic mice (5XFAD), created by Oakley et al. (2006), express 2 PSEN and 3 APP mutations and overexpress Aβ42 (Eimer and Vassar, 2013). Intraneuronal Aβ42 is identifiable at 1.5 months and plaque formation is observed by 2 months (Eimer and Vassar, 2013; Oakley et al., 2006; Richard et al., 2015; Bader et al., 2023; Gail Canter et al., 2019). Cognitive deficits have been seen by 3–6 months of age in 5xFAD Het mice (Jawhar et al., 2012; Oakley et al., 2006), although one research group reports spatial memory deficits in the Morris water maze as early as 1 month of age (Tang et al., 2016; Wu et al., 2018). Mitochondrial dysfunction, based on RNA-SEQ changes, shows up as early as 7 weeks (Kim et al., 2012). Given that 5XFAD mice express 2 PSEN mutations, early NMDAR hyperexcitability and calcium dysregulation are likely.

Impaired energy metabolism is one of the earliest and most consistent features of AD (Yu et al., 2022; Mosconi, 2013), which is closely linked to mitochondrial dysfunction. Electron microscopy has revealed specific mitochondrial alterations in AD brains, including size heterogeneity, disrupted or absent cristae, and elongated interconnected organelles. Oxidative stress markers closely linked to loss of synaptic proteins in MCI and pre-AD patients indicate that mitochondrial dysfunction plays a central role in AD pathology.

The current study addressed the hypothesis that there are early subcellular changes in the 5xFAD mouse model that occur before significant amyloid plaque deposition or major cognitive deficits. This hypothesis is grounded in the convergence of: (1) evidence for pre-plaque synaptic and anatomical changes across multiple AD models; (2) PSEN1 mutation-driven NMDAR hyperexcitability mechanistically linked to calcium and potentially mitochondrial stress; and (3) the 5xFAD model’s unique combination of PSEN and APP mutations enabling investigation of these interactions during early Aβ production. This study determined the age of onset of significant transgene expression (15 days) and plaque accumulation (4 months) in the 5xFAD mouse model. Electrophysiological recording confirmed AMPAR and GluN2B hyperexcitability at 1 month of age. Mitochondrial morphology showed pronounced structural damage at this young age in hippocampal synaptic processes and transgene impact on dendritic morphology revealed a hippocampal subregional susceptibility substantiated by spatial transcriptomics. All findings were correlational and descriptive; however, our results provide the foundation for future experiments to establish causal hierarchies, which were beyond the scope of this current study.

## 2 Materials and methods

### 2.1 Animals

The 5xFAD mouse model, B6.Cg Tg(APPSwFlLon,PSEN1*M146L*L286V)6799Vas/Mmjax, RRID:MMRRC_034848-JAX, was obtained from MMRRC at The Jackson Laboratory, an NIH-funded strain repository, and was donated to the MMRRC by Robert Vassar, Ph.D., Northwestern University. 5xFAD Het male mice were bred with female C57BL/6J mice (JAX Labs) to produce non-transgenic littermates (WT) or 5xFAD heterozygous (5xFAD Het) mice. Mice were fed *ad libitum* with standard rodent chow on a 12-h light/dark cycle in AALAC-approved space at Oregon State University. All experiments were approved by the Oregon State University IACUC (IACUC-2019-0043, IACUC-2020-0082, ACUP4921). Mice used for electron microscopy (EM), Golgi analysis and spatial transcriptomics were sacrificed using isoflurane and decapitation, and brains were quickly removed for processing. Mice used for electrophysiology and immunohistochemistry were euthanized as described below for electrophysiology. WT and 5xFAD Het mice were collected at 15 days or 1, 2, 3 or 4 months of age for immunohistochemistry and at 1 month of age for all other studies. A completed analysis of female genotype differences revealed glutamatergic receptor hyperactivity in electrophysiology experiments. The subsequent exploratory experiments for other outcomes used combined sexes to reduce animal waste.

### 2.2 Genotyping

Tissue from ear punches were collected at ∼15 days of age. Genotyping was performed by Transnetyx, Inc., or in-house using the protocol and primer sequences provided by Jackson Laboratories^1^. WT animals have a band at 219 bp only; 5xFAD Het animals have an additional band at 129 bp.

### 2.3 QPCR: transgene expression

To assess the onset of mRNA expression of the transgene, small pieces of brain tissue (∼10 mg) were used for QPCR on N(WT) = 3 (2Female (F)/1Male (M)) and N(Het) = 3 (2F/1M) per age group (0.5 and 1 month) animals (male and female combined). Total RNA was extracted using the Qiagen RNeasy Lipid Tissue Mini Kit (Cat. # 74804). cDNA was synthesized using a Thermo Fisher SuperScript IV kit (Cat. # 18091050). Samples were analyzed using TaqMan Gene Expression Master Mix and a StepOne Plus RT-PCR machine. Protocol and primer sequences provided by Jackson Laboratories (see Footnote 1). GAPDH (Mm99999915_g1) was used for normalization. Due to differences in primer efficiency, the Pfaffl method was used; all samples were normalized to cortical tissue from a 0.5-months-old WT animal.

### 2.4 Immunohistochemistry

β-amyloid IHC was performed on hippocampal slices from male and female WT and 5xFAD mice at 0.5-N(WT) = 10 (6F/4M) & N(Het) = 7 (4F/3M), 1-N(WT) = 6 (4F/2M) & N(Het) = 6 (3F/3M), 2-N(WT) = 7 (3F/4M) & N(Het) = 7 (3F/4M), and 4-N(WT) = 8 (5F/3M) & N(Het) = 7 (4F/3M) months of age. Animals were euthanized and slices prepared as described below for electrohysiology experiments. Slices were treated with: PBS-T washes; 0.3% H_2_O_2_ peroxidase block; 3%–5% goat serum block; 1:500 rabbit anti-beta amyloid antibody (abcam ab2539, overnight); biotin-conjugated secondary antibody (1:500 goat anti-rabbit IgG, 2 h); Vectastain ABC Kit (1:800, 1 h); and DAB/H_2_O_2_ (7 min). After dehydration and coverslipping, images were acquired at 200× from three areas centered on the CA1 stratum radiatum and lacunosum/moleculare from 3 slices (9 images/mouse). ImageJ was used for quantifying percent area stained above threshold (max brightness 170, threshold 70). All analyses were performed blind. Statistical analysis was via three-way ANOVA (*p* < 0.05) with within-age *post hoc* comparisons to assess genotype differences at each individual timepoint.

### 2.5 Electrophysiology

**FIGURE 1:**
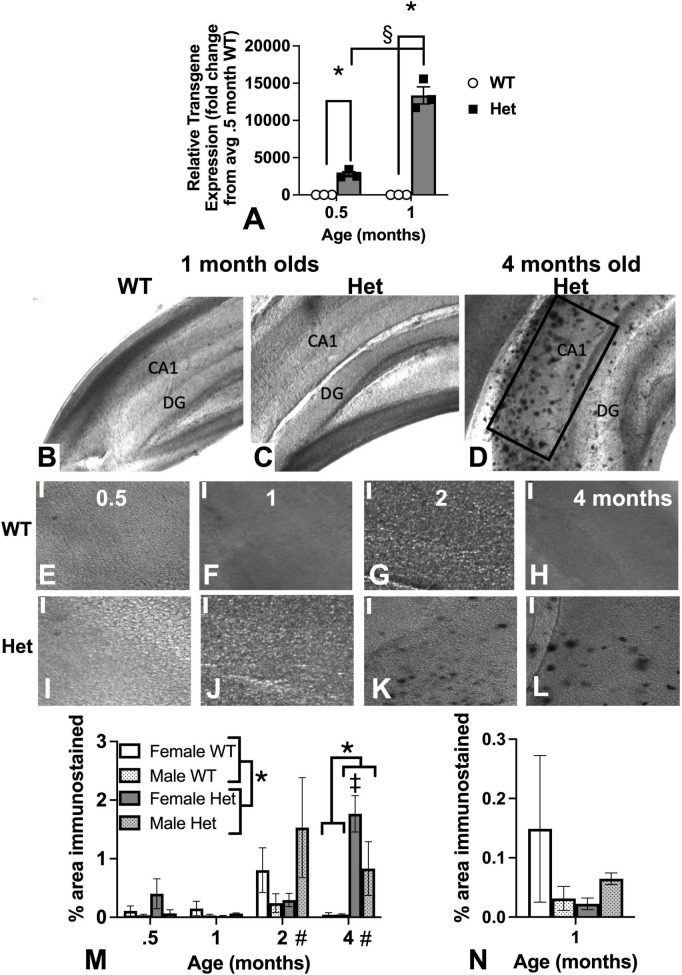
Genotypic differences in glutamate receptor activity at 1 month in CA1. **(A)** I/O session timeline and drug delivery. **(B)** Example individual I/O curves for the AMPAR component. **(C)** Example I/O curve demonstrating pharmacological isolation of the GluN2B component (Ro 25-6981-sensitive). **(D)** Averaged AMPAR component responses: female 5xFAD Hets showed higher AMPAR responses than WT. **(E)** Averaged GluN2B component responses: female 5xFAD Hets showed higher GluN2B responses than WT. Extra sum-of-squares F Test, non-linear regression. N(WT female) = 4, N(Het female) = 7. Symbols = mean, bars = SEM.

For electrophysiology experiments, 1-month-old female WT (*N* = 4) and 5xFAD Het (*N* = 7) mice were anesthetized (ketamine 100 mg/kg + xylazine 20 mg/kg IP, then isoflurane) and rapidly perfused with ice-cold carboxygenated aCSF (NaCl 124 mM, KCl 2 mM, KH_2_PO_4_ 1.25 mM, MgSO_4_ 2 mM, Glucose 10 mM, NaHCO_3_ 26 mM, no Ca^2+^). Coronal slices (300 μm) were prepared by vibratome, recovered at 32°C for 30 min, then at room temperature for 2.5 h. Slices were placed onto a 16-electrode Med 64 Quad II probe (AutoMate Scientific). Schaffer collaterals were stimulated and fEPSPs recorded in CA1 stratum radiatum. Slices were perfused with carboxygenated (95% O_2_ + 5% CO_2_) aCSF with 2 mM CaCl at 2 mL/min at 32°C. Sequential antagonists were applied: DNQX (30 μM); DNQX, Picrotoxin (10 μM) and low Mg (0.5 mM); and Ro 25-6981 (4 μM; Figure 1A). Input (fiber volley)/output (fEPSP; I/O) curves were obtained after each drug stabilization period (≥20 min). An I/O consisted of stimulations from 10 to 60 pAmps in 5 pAmp increments every 20 s. Each stimulation level from post-treatment I/O curves were subtracted from the same stimulation level from a prior I/O curve, and the differences were then expressed as the I/O curve for a specific receptor or subunit component of the fEPSP (Figure 1B). Statistical analysis used extra sum-of-squares F Test comparison of non-linear regression curve fits (Prism).

### 2.6 Electron microscopy

Heterozygous 5xFAD mice (*N* = 6 mice; Mitochondria analyzed per mouse = 61, 68, 85, 99, 113 and 114) and WT littermates (*N* = 3: Mitochondria analyzed per mouse = 74, 94, 102) at 1 month of age were used. Welch’s *t*-tests were used for all comparisons to accommodate the unbalanced design and unequal variances. The WT animals were vehicle controls from a concurrent treatment study, receiving PBS IP every 3 days from postnatal day 15–30. Sex was not documented. Hippocampi were immersion-fixed in modified Karnovsky’s fixative, T-O-T-O (1.5% potassium ferrocyanide + 2% osmium tetroxide) post-stained, incubated in uranyl acetate and lead aspartate, dehydrated through a graded ether-acetone series, and embedded in araldite resin. Ultrathin sections (∼5 μm) were imaged on a FEI Helios Nanolab 650 in STEM mode. Images containing synaptic vesicles, post-synaptic densities, and “railroad track” membranes were selected for analysis. Mitochondrial counts were performed manually and size was quantified using FIJI. Cristae integrity was scored blindly by two investigators: 1 = intact, 2 = partial disruption or denudation but retaining some intact structures, 3 = widespread collapse or near-complete loss of structure.

### 2.7 Golgi stain and analyses

Whole brains from 1-month-old WT and 5xFAD Het (male and female combined) mice were stained using the Golgi-Cox Rapid Fast Stain Kit (FDNeurotechnologies, PK401) and sectioned at 100 μm/slice. Neurons were contributed by N(WT) = 6 (3F/3M) and N(Het) = 6 (3F/3M) mice. For Sholl analysis, totals of 30, 24, and 30 neurons were used per group from CA1, CA3, and DG regions, respectively. Z-stacks were taken on a Leica DM6000 (20× CA1, 10× CA3, 40× DG), processed in FIJI (Schindelin et al., 2012), and traced using Simple Neurite Tracer (Longair et al., 2011). Sholl analysis calculated intersections every 20 μm from the soma; total neuron length and area under the curve were also calculated. For spine density, 30 neurons per group from CA1 and CA3 (basal dendrites) and 24 from DG (apical dendrites) were analyzed using 60× images. All analyses were performed blind.

### 2.8 Spatial transcriptomics

1-month-old 5xFAD Het (*N* = 4 (1F/3M) and WT (*N* = 4 (2F/2M) littermates (male and female combined) were used. Cryostat sections (6 μm) through the dorsal hippocampus were processed by Nanostring, Inc., by treating them with indexing UV-photocleavable oligo-labeled cDNA probes using the GeoMx Mouse Whole Transcriptome Atlas Mouse RNA for Illumina Systems (Catalog# GeoMx NGS RNA WTA Mm). Sections were stained for NeuN, GFAP, Iba1, and DNA; regions of interest (ROIs: CA1, CA3, DG) were outlined. UV light was used to collect oligo-barcodes within each outlined subregion, which were quantified with nCounter (Nanostring, Inc.). Sample data, separated into subregions, were grouped via t-SNE (van der Maaten and Hinton, 2008) or UMAP (McInnes et al., 2020) for data visualization. One female WT mouse was excluded (no transcript detection), giving N(WT) *N* = 3 (1F/2M) and N(Het) *N* = 4 (1F/3M). Raw counts were analyzed by NOISeqBio (Tarazona et al., 2015) with TMM normalization, 1000 permutations, |FC| > 1.5, and *p* < 0.05 thresholds. Gene Set Enrichment Analysis (GSEA) used the UC San Diego and Broad Institute software (Subramanian et al., 2005; Mootha et al., 2003) and Molecular Signatures Database (Liberzon et al., 2011, 2015). Genotype differences in Reactome (Gillespie et al., 2022) and Gene Ontology (Ashburner et al., 2000; Gene Ontology et al., 2023) database pathways were analyzed in each subregion, using 1000 gene set permutations for each comparison and FDR < 0.25. Data were visualized with Matplotlib (Hunter, 2007) and ggplot2 (Wickham, 2016) with a mouse brain single-cell reference (Yao et al., 2021). Differential expression by cell type between genotypes used a reverse deconvolution linear regression. Mitochondrial gene panel regulation was assessed by Chi-Square test. Functional biological impact was assessed via GSEA (Korotkevich et al., 2019) with adjusted *p*-value < 0.25.

### 2.9 Statistical analyses

Statistical analyses were performed using GraphPad Prism 9, R studio, and G*Power. QPCR: 2-way ANOVA and Fisher’s LSD. Electrophysiology: non-linear regression curve fit and extra sums-of-squares F test. EM: Welch’s *t*-test (selected for unbalanced group sizes and unequal variances). Sholl analysis: repeated measures ANOVA with multiple testing correction. β-amyloid staining, transgene expression, neuronal length, area under the curve, and spine density: ANOVA with *post hoc* analysis. Within-age *post hoc* comparisons for beta-amyloid staining were performed to assess genotype differences at each timepoint. All graphs made using GraphPad Prism.

## 3 Results

### 3.1 5 xFAD transgene was detectable at 0.5 months of age

**FIGURE 2:**
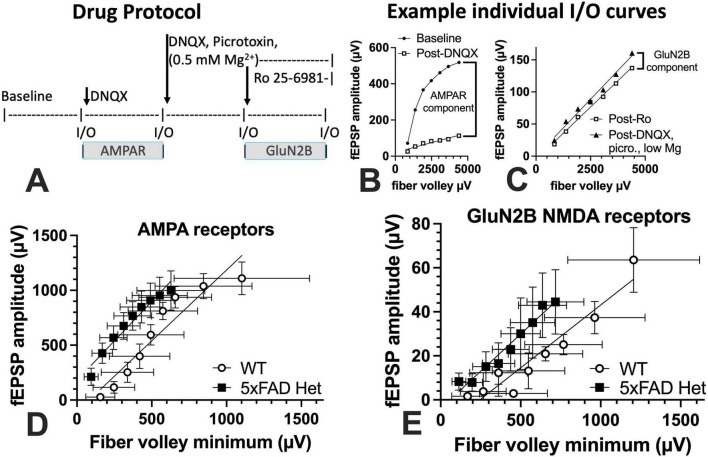
5xFAD transgene expression and β-amyloid immunostaining. **(A)** Measurement of 5xFAD transgene expression by qPCR in enriched CA1 tissue from 0.5 to 1 month old WT and 5xFAD Hets. Hets had more transgene expression than WT (*) at both ages. 1 month old Hets had more than 0.5 month Hets (§). N(WT) = 3 (2F/1M), N(Het) = 3 (2F/1M) per age group. **(B–D)** Low magnification images of β-amyloid immunostaining from 1 month old WT **(B)** and Het **(C)** and 4 months old Het **(D)** hippocampal slices. Three sample images were obtained from within the general area indicated by the rectangle in panel **(D)**, centered on CA1 stratum radiatum and laconosum/moleculare. **(E–L)** Representative higher magnification sample images from WT **(E–H)** and Hets **(I–L)** at 0.5 **(E,I)**, 1 **(F,J)**, 2 **(G,K)**, and 4 months of age **(H,L)**. Upper left vertical bar = 20 μm. **(M)** Graph showing 5xFAD Het mice had greater β-amyloid immunostained % area overall and within 4 months olds (*) than WT and female Hets had a larger % area at 4 months (‡) compared to female WT of the same age. Mice at 2 and 4 months had more area stained than 1 month olds (#). **(N)** Enlarged view of 1 month old data. Mice were 0.5- (N(WT) = 6 female(F)/4 male(M); N(Het) = 4F/3M), 1- (N(WT) = 4F/2M; N(Het) = 3F/3M), 2- (N(WT) = 3F/4M; N(Het) = 3F/4M), and 4- (N(WT) = 5F/3M; N(Het) = 4F/3M) months of age. ANOVA and Fisher’s LSD.

There was a significant main effect of Age (*p* < 0.0001) and Genotype (*p* < 0.0001) and a significant Age × Genotype interaction (*p* < 0.0001) on transgene expression (Figure 2A). Transgene expression was significantly higher at 0.5 (*p* = 0.01) and 1 (*p* < 0.0001) months of age in 5xFAD Het mice than WT. The 5xFAD Het mice at 1 month had significantly higher expression than at 0.5 months (*p* < 0.0001; Figure 2A).

### 3.2 Significant β-amyloid plaque burden in 5xFAD Het mice was not evident until 4 months of age

Figures 2B–L shows representative β-amyloid immunostaining of hippocampal slices (Figures 2B–D) and higher magnification sample images (Figures 2E–L) from different ages and genotypes. There was a significant main effect of Age (*p* = 0.0059) and Genotype (*p* = 0.0067), but not Sex (*p* = 0.55), on percent area of β-amyloid immunostaining (Figures 2B–N). A significant Age × Genotype × Sex interaction was observed (*p* = 0.02). Within-age *post hoc* comparisons confirmed no significant genotype differences at 0.5 (*p* = 0.552), 1 (*p* = 0.851), or 2 months (*p* = 0.117); a significant genotype difference was present only at 4 months (*p* = 0.0001; Figures 2B–N). 5xFAD Hets had significantly more percent area of staining than WT overall and within 4 month olds (*p* = 0.0001; Figure 2M). Female 4-months-old Hets also had more area stained than female 4-months-old WT (*p* < 0.0001; Figure 2M). The 2 and 4 months old mice had larger percent area than 1 month olds (*p* = 0.038–0.049; Figure 2M). At 2 months, male Het animals showed a trend toward higher staining relative to male WT – the opposite directionality from the female-predominant pattern at 4 months – consistent with the significant Age × Genotype × Sex interaction term (*p* = 0.02). This sex-dependent trajectory at early ages is an intriguing finding that warrants future investigation with adequately powered sex-stratified cohorts. The visible staining in WT animals at 1–2 months (Figures 2M, N) was attributed to non-specific DAB background signal rather than true Aβ plaque formation, as this pattern lacked focal plaque morphology and did not differ statistically from other WT at the applied threshold.

### 3.3 Glutamatergic hyperactivity was present at 1 month of age

Glutamate receptor function was assessed in hippocampal slices from 1 month old female WT and 5xFAD Het mice. Slices were stimulated over the Schaffer collateral axons and recorded in the stratum radiatum of the CA1 region. Sequential inhibitory drugs were applied to determine the relative contribution of AMPA receptors and GluN2B subunits of NMDA receptors (Figures 1A–C). Female 5xFAD Het animals exhibited higher AMPA receptor (Figure 1D; *p* < 0.0001) and GluN2B subunit (Figure 1E; *p* = 0.024) responses for a given fiber volley input than WT at 1 month of age.

### 3.4 Mitochondrial damage was increased at 1 month of age

**FIGURE 3:**
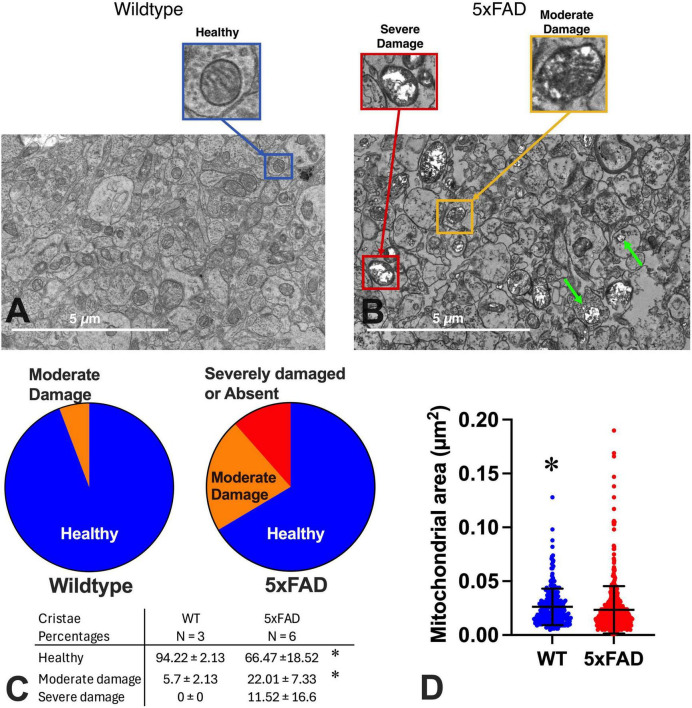
Representative EM images and quantitative assessment of cristae integrity in hippocampal synaptic mitochondria of WT and 5xFAD Het mice. **(A)** WT synaptic region with intact mitochondrial cristae. **(B)** 5xFAD Het synaptic region with disrupted mitochondrial morphology. Insets show examples of healthy (blue boxes), moderately damaged (yellow boxes), and severely damaged (red boxes) mitochondria. Green arrows indicate reduced sized mitochondria. Bars = 5 μm. **(C)** Cristae integrity quantification. >90% of WT mitochondria were healthy. 5xFAD showed significantly higher moderate disruption (*p* = 0.002) and fewer healthy mitochondria (*p* = 0.0139). Table: mean ± SD for each damage category. **(D)** 5xFAD mitochondria had a wider range of areas and an average size that was smaller than WT (*p* = 0.0448). *p < 0.05 between genotypes. Mean ± SD. N(WT) = 3 mice (Mitochondria numbers analyzed per mouse = 74, 94, 102) N(5xFAD Het) = 6 mice (Mitochondria numbers analyzed per mouse = 61, 68, 85, 99, 113 and 114). Sex was not documented.

Ultrastructural analysis of hippocampal synaptic mitochondria was performed using STEM in 1-month-old 5xFAD heterozygous mice and WT littermates. Only mitochondria at synaptic regions – identified by synaptic vesicles, post-synaptic densities, and “railroad track” membranes – were included (Figures 3A, B).

In WT mice, synaptic mitochondria exhibited uniform spherical morphology, consistent size, and homogeneous electron density. Cristae were densely packed (Figure 3A). In contrast, 5xFAD mitochondria displayed pronounced abnormalities: irregular shapes, variable sizes, increased membrane electron density, and fragmented or vacuolated profiles (Figure 3B). Mitochondrial number per synaptic image field did not differ significantly between genotypes [*t*(6.177) = 0.000, *p* > 0.9999; not shown].

Semi-quantitative evaluation of cristae integrity revealed significant differences. In WT hippocampi, >90% of mitochondria were healthy, with few showing moderate or severe damage (Figure 3C). In 5xFAD hippocampi, ∼45% of mitochondria were moderately or severely damaged. The proportion of healthy mitochondria was significantly lower in 5xFAD mice [*t*(5.259) = 3.622, *p* = 0.0139]. Moderate damage was significantly higher in 5xFAD [*t*(6.379) = 5.018, *p* = 0.002] and there was a trend for a higher proportion of severely damaged mitochondria [*t*(5) = 1.7, *p* = 0.1499]. Damaged mitochondria often exhibited shortened or absent cristae and vacuolated inner compartments. The 5xFAD mitochondria showed a wider range of areas and, on average, were significantly smaller in average area than WT [Figure 3D; *t*(676.4) = 2.01, *p* = 0.0448]. Note that mitochondrial area represents a 2D cross-sectional measure from single-plane STEM images; orientation and sectioning bias can influence apparent cross-sectional area, and these values should be interpreted as a relative indicator rather than absolute volume. Collectively, these findings demonstrate that synaptic mitochondria in young 5xFAD mice displayed substantial ultrastructural abnormalities indicative of early mitochondrial distress, preceding overt amyloid pathology.

### 3.5 Regional differences in dendritic complexity, length, and spine density at 1 month of age

**FIGURE 4:**
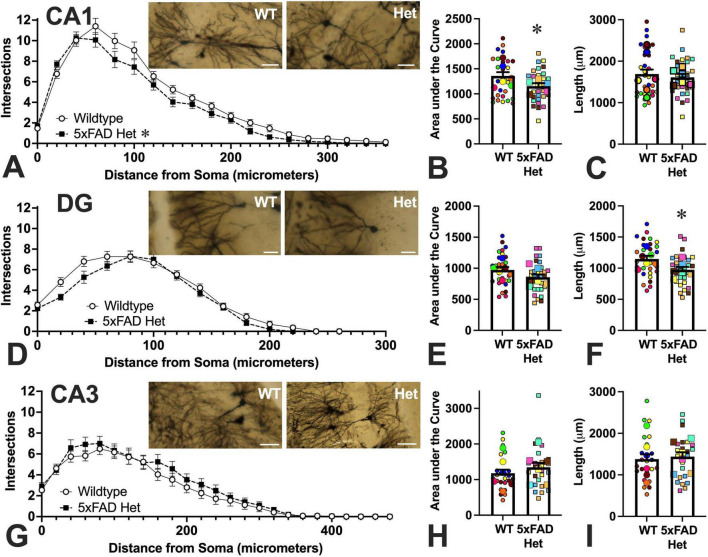
Sholl analysis of dendritic arborization **(A,D,G)**, area under the curve **(B,E,H)**, and dendritic length **(C,F,I)** for CA1 pyramidal **(A–C)**, DG granule cell **(D–F)** and CA3 pyramidal **(G–I)** neurons from 1-month-old WT and 5xFAD Hets. Inset Golgi-Cox images in panels **(A,D,G)** (Bars = 25 μm). 5xFAD Het CA1 neurons had fewer Sholl intersections **(A)** and reduced AUC **(B)**. DG granule cells showed shorter dendritic length **(F)**. In SuperPlot graphs, small symbols indicate individual neuronal data, larger symbols indicate animal means. Colors differentiate animals. Sholl: repeated measures ANOVA (CA1, CA3) or mixed effects ANOVA (DG). Area, length: unpaired *t*-test. Mean ± SEM. *Indicates *p* < 0.05 for difference from WT. N(WT) = 6 mice (3F/3M), N(Het) = 6 mice (3F/3M); 30 neurons/group (CA1 & DG), 24 neurons/group (CA3).

**FIGURE 5:**
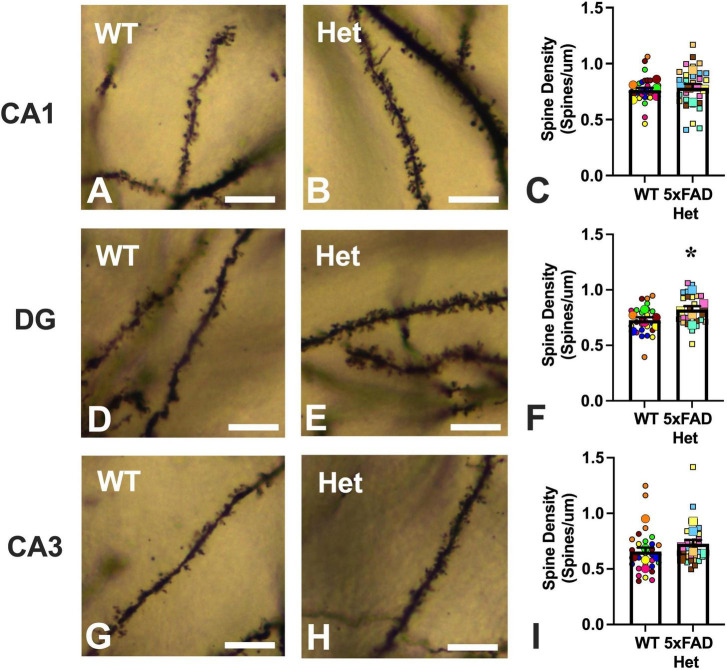
Spine density from dendrites of CA1 pyramidal **(A–C)**, DG granule cell **(D–F)**, and CA3 pyramidal **(G–I)** neurons. Representative images from WT **(A,D,G)** and 5xFAD Hets **(B,E,H)**. Spine density graphs from CA1 **(C)**, DG **(F)**, and CA3 **(I)**. 5xFAD Hets had significantly higher spine density in DG **(F)**. In SuperPlot graphs, small symbols indicate individual neuronal data, larger symbols indicate animal means. Colors differentiate animals. Welch’s *t*-test. Mean ± SEM. *Indicates *p* < 0.05 for difference from WT. N(WT) = 6 mice (3F/3M), N(Het) = 6 mice (3F/3M); 30 neurons (CA1 & CA3), 24 neurons (DG). Bars = 10 μm.

For CA1 pyramidal cells, there was a significant main effect of Genotype (*p* = 0.023) on Sholl analysis intersections (Figure 4A) and area under the curve analyses (*p* = 0.034; Figure 4B), but none for dendritic length (*p* = 0.55; Figure 4C). 5xFAD Het CA1 pyramidal cells had fewer intersections and reduced area under the curve than WT. There was no significant main effect of Genotype on intersections (*p* = 0.063; Figure 4D) or area under the curve (*p* = 0.077; Figure 4E) for DG granule cells, but there was a significant reduction in dendritic length in 5xFAD Het DG granule cells (*p* = 0.014; Figure 4F). There was no significant effect of genotype on intersections (*p* = 0.31; Figure 4G), area under the curve (*p* = 0.30; Figure 4H) or dendritic length (*p* = 0.70; Figure 4I) for CA3 pyramidal cells. All regions showed significant Distance-from-soma effects (all *p* < 0.0001). There were no significant genotypic effects on spine density on CA1 pyramidal cell basal dendrites (*p* = 0.56; Figures 5A–C). There was a significant increase in spine density on 5xFAD Het apical dendrites in the DG (*p* = 0.02; Figures 5D–F) vs. WT. No genotypic differences in spine density were found on CA3 pyramidal cell basal dendrites (*p* = 0.16; Figures 5G–I).

### 3.6 Spatial transcriptomics showed differential effects of genotype on hippocampal subregions at 1 month of age

**FIGURE 6:**
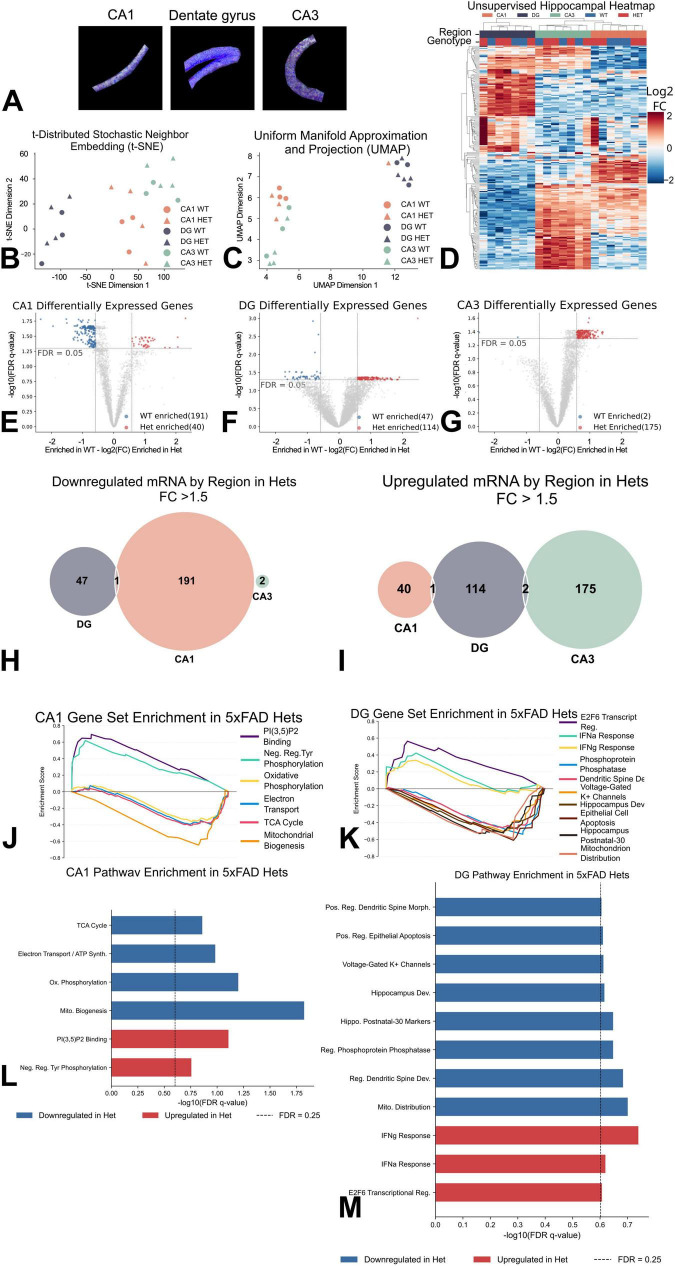
Spatial transcriptomics of hippocampal subregions. **(A)** Representative hippocampal ROI images stained with NeuN, GFAP, Iba1, and DNA confirming anatomical subregion boundaries for CA1, CA3, and DG. **(B,C)** tSNE and UMAP showing subregion separation. **(D)** Hierarchical clustering of global log-fold changes by genotype and subregion. Transcript information available in Supplementary Figure 1. **(E–G)** Volcano plots for CA1 **(E)**, DG **(F)**, CA3 **(G)** (| FC| > 1.5, FDR < 0.05). **(H,I)** Venn diagrams of DEG overlap. **(J–M)** GSEA enrichment plots and significance for CA1 **(J,L)** and DG **(K,M)**. N(WT) = 3 (1F/2M) and N(Het) = 4 (1F/3M).

Figure 6A shows representative images of hippocampal ROIs. Sections were stained for NeuN, GFAP, Iba1, and DNA to unambiguously delineate CA1, CA3, and dentate gyrus subregions and confirm appropriate ROI boundaries in both WT and 5xFAD Het animals.

Both tSNE and UMAP (Figures 6B, C) and hierarchical clustering (Figure 6D) showed grouping along subregions but not genotype, providing rationale for subregion-level differential gene expression analysis. In CA1, 191 transcripts were decreased and 40 increased in 5xFAD Hets vs. WT (Figures 6E, H, I). In DG, 47 were decreased and 114 increased (Figures 6F, H, I). In CA3, 2 were decreased and 175 increased (Figures 6G, H, I). Subregion transcript changes showed little overlap (Figures 6H, I).

Gene Set Enrichment Analysis revealed differentially regulated pathways in CA1 (Figures 6J, L) and DG (Figures 6K, M), but not CA3. CA1 from 5xFAD Hets showed higher mRNA for negative regulation of tyrosine phosphorylation and PI-3,5-bisphosphate binding; downregulated gene sets included citric acid cycle, electron transport and ATP synthesis, oxidative phosphorylation, and mitochondrial biogenesis (Figure 6L and Supplementary Table 1). It should be noted that several GSEA *q*-values are in the range 0.10–0.25 (Supplementary Table 2), which, while meeting the FDR < 0.25 threshold, are more marginal than the mitochondrial biogenesis *q*-value (FDR = 0.015); interpretive weight should be placed accordingly. DG upregulated gene sets included transcriptomic signatures consistent with upregulation of interferon alpha and gamma response pathways. Whether this transcriptomic signature reflects active microglial or astrocyte activation at this age was not directly tested by IHC in this study; future studies with quantified Iba1+ microglial morphology and GFAP+ astrocyte reactivity data would be needed to confirm a neuroinflammatory phenotype. Downregulated DG gene sets included voltage-gated potassium channels, phosphatase activity regulation, and mitochondrion distribution (Figure 6M and Supplementary Table 1).

### 3.7 Single cell deconvolution suggests CA1 pyramidal neurons are the most vulnerable population to mitochondrial changes

**FIGURE 7:**
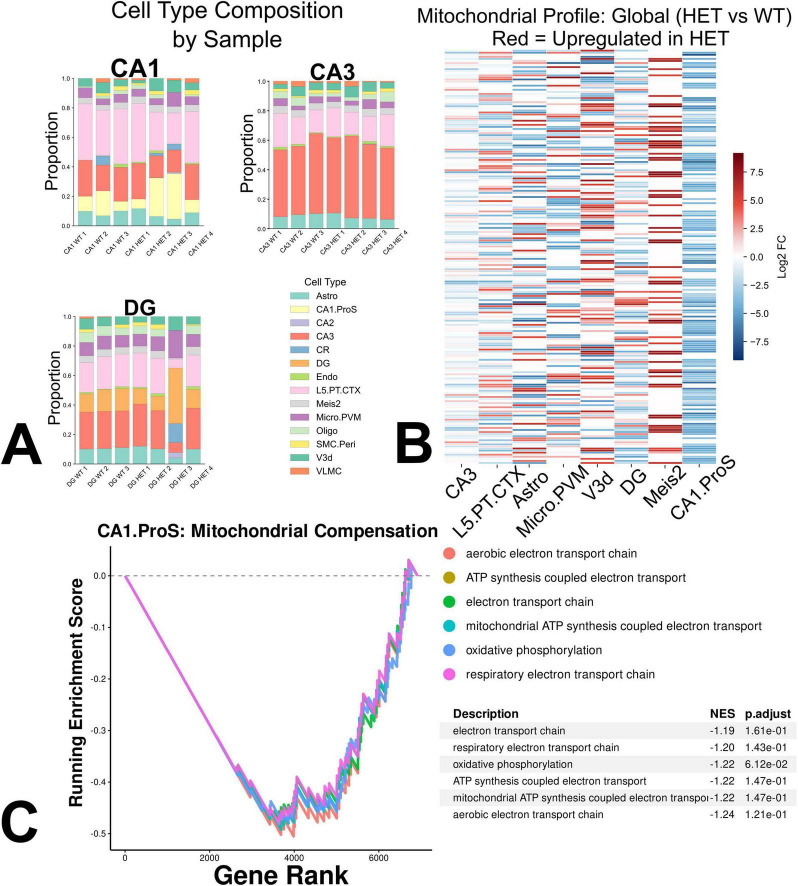
CA1 pyramidal neurons exhibited coordinated mitochondrial downregulation. **(A)** Cell type composition from spatial transcriptomic deconvolution confirming consistent cellular architecture between WT and HET. **(B)** Heatmap of mitochondrial gene Log2FC in HET vs. WT. Red = upregulated; Blue = downregulated. CA1.ProS neurons show coordinated downregulation. Statistical analysis confirms CA1.ProS as primary driver (χ^2^ = 219.54, *p* < 0.0001), with significantly higher proportion of downregulated transcripts (Fisher’s Exact, *p* < 0.0001). Transcript information available in Supplementary Figure 2. **(C)** GSEA of CA1.ProS profile showing negative enrichment (NES = –1.24) for “Oxidative Phosphorylation” and “Aerobic Electron Transport Chain,” confirming enrichment among most downregulated genes in HET CA1.ProS neurons. N(WT) = 3 (1F/2M) and N(Het) = 4 (1F/3M).

To determine whether mitochondrial defects were ubiquitous or cell-type specific, spatial deconvolution reconstructed the cellular architecture of the hippocampus. CA1.ProS neurons, CA3 neurons, and DG granule cells localized as expected (Figure 7A). No significant shifts in cell-type proportional abundance were observed between genotypes, indicating no overt neurodegeneration at this timepoint.

Analysis of cell-type specific gene expression revealed a profound metabolic divergence. A heatmap of mitochondrial transcripts showed that while most cell types displayed heterogeneous or neutral expression changes, CA1.ProS neurons exhibited a striking, coordinated downregulation across the majority of the panel (Figure 7B). The distribution of up- vs. down-regulated mitochondrial genes was significantly dependent on cell type (χ^2^ = 219.54, *p* < 0.0001). CA1.ProS neurons were the primary driver of this variance (*Z* = 10.79, *p* < 0.0001), exhibiting a significantly higher proportion of downregulated transcripts. Fisher’s Exact test confirmed CA1.ProS neurons were significantly more likely to exhibit mitochondrial downregulation compared to all other cell types combined (*p* < 0.0001).

Gene Set Enrichment Analysis of the CA1.ProS expression profile revealed a robust negative enrichment for canonical energy production pathways including “Aerobic Electron Transport Chain” and “Oxidative Phosphorylation” (Figure 7C). The normalized enrichment score (NES = −1.24) confirms these gene sets are enriched among the most downregulated genes in HET CA1.ProS neurons relative to WT. The specific suppression of these pathways in HET CA1 neurons–but not CA3 neurons or glia–is consistent with a localized compensatory or pathological mechanism. These transcriptomic observations are correlational; the functional consequences for mitochondrial respiration in CA1 pyramidal neurons remain to be directly tested by synaptosomal respirometry or genetically encoded metabolic sensors in future studies.

## 4 Discussion

Initially, research and treatments focused on insoluble Aβ plaques as the initiating factor for AD (Krafft et al., 2022), but newer evidence suggests these may represent responses to injury rather than inciting causes (Mondragon-Rodriguez et al., 2010). Evidence is growing that Aβ42 or Aβ40 oligomers can trigger neurodegeneration and cognitive deficits (Tolar et al., 2021; Krafft et al., 2022). In this study, the transgene was expressed by 15 days of age but plaque accumulation was not statistically significant until 4 months. Despite this, 5xFAD Hets showed glutamate receptor hyperactivity, mitochondrial morphological alterations and associated RNA changes, and attenuation in dendritic complexity at 1 month of age, with the CA1 region showing the greatest changes. The parallel occurrence of glutamatergic hyperexcitability, mitochondrial disruption, and dendritic changes at 1 month of age is consistent with a connected pathological process, but the mechanistic hierarchy among these changes remains to be established. The findings reported here provide the foundation for future studies designed to establish a cause-and-effect sequence of events between amyloid expression, NMDAR activity, and mitochondrial function.

### 4.1 Plaque timeline

It is important to contextualize the plaque timeline against prior published reports on 5xFAD mice. Eimer and Vassar (2013) and Oakley et al. (2006) reported intraneuronal Aβ42 at 1.5 months and plaque formation observed by 2 months. In the present study, statistical difference from WT significance was reached only at 4 months. Several factors likely explain this: (1) our colony is maintained on a pure C57BL/6J background vs. the mixed B6/SJL background of the original descriptions; however, we observed plaques at 2 months of age, similar to others using the C57BL/6J background (Richard et al., 2015; Bader et al., 2023; Gail Canter et al., 2019); (2) our percent-area threshold quantification may differ from visual plaque counting; and (3) the difference between observing plaques (Richard et al., 2015; Bader et al., 2023; Gail Canter et al., 2019) and statistical difference in area of staining. Our qPCR data confirmed robust transgene expression by postnatal day 15 establishing that the delayed timeline reflects late plaque nucleation rather than absent transgene activity.

### 4.2 Glutamate receptor hyperactivity

The 1-month-old 5xFAD Hets showed increased GluN2B subunit-related synaptic responses. This is consistent with PSEN1 mutant mice, which show transient NMDAR hyperexcitability at 3–5 months (Auffret et al., 2009, 2010; Dewachter et al., 2008) and specifically with increased GluN2B subunit expression in PS1A246E mutants (Dewachter et al., 2009). The two PSEN1 mutations in 5xFAD mice may explain the earlier onset compared with single PSEN1 mutants. NMDAR hyperactivity in PSEN1 mutants is associated with enhanced early LTP but reduced late LTP (Auffret et al., 2010), suggesting it is not beneficial for long-term synaptic plasticity. Although NMDARs are normally good for initiating memories and synaptic plasticity (Morris et al., 1986; Alessandri et al., 1989; Butelman, 1989; Heale and Harley, 1990; Mondadori et al., 1989), overstimulation by glutamate leads to calcium overload-related excitotoxicity (Choi, 1992). Soluble Aβ can increase GluN2B-related responses linked to LTP inhibition (Li et al., 2011), cause synaptic depression via non-ionotropic NMDA receptor functions (i.e., not via its ion channel) (Kessels et al., 2013), and interactions between Aβ and GluN2B-containing receptors are associated with increased mitochondrial calcium and membrane depolarization (Ferreira et al., 2015).

The 5xFAD Hets also showed elevated AMPAR activity at 1 month, distinguishing them from PSEN1 mutant mice which show no AMPAR changes (Auffret et al., 2009, 2010; Dewachter et al., 2008). Aβ oligomers can increase both NMDAR and AMPAR calcium currents in organotypic cultures (Alberdi et al., 2010). Aβ has been shown to increase synaptic glutamate by enhancing astrocytic glutamate release (Talantova et al., 2013) or decreasing transporter-mediated uptake (Li et al., 2009, 2011). Excess synaptic glutamate may explain the elevated responses in both AMPA and NMDA receptors in the 1-month-old 5xFAD Hets.

The co-occurrence of NMDAR and AMPAR hyperactivity with calcium dysregulation provides context for the mitochondrial observations described below. NMDAR hyperactivity in PSEN mutants has been linked to ER calcium buffering deficits (Costa et al., 2012; Schneider et al., 2001) and potentially to mitochondrial calcium buffering deficits (Stanika et al., 2009; Ryan et al., 2020; Mustaly-Kalimi et al., 2025). Whether the co-occurrence of these phenotypes in 5xFAD Hets reflects a causal or merely parallel relationship remains to be determined. There is evidence that neuronal hyperactivity may be relevant to the development of AD, as humans with mild cognitive impairment, a preclinical stage of AD, show higher activation of brain regions while performing cognitive tasks than those with normal cognition (Dickerson et al., 2005; Foster et al., 2018; Mormino et al., 2012).

### 4.3 Mitochondrial injury and early bioenergetic compromise

Synaptic mitochondria in 1-month-old 5xFAD Hets displayed pronounced ultrastructural damage – cristae disruption, vacuolization, increased electron density – without net loss in mitochondrial number (Figure 3). The EM findings were obtained from hippocampal synaptic fields without subregion specification; the subregion-specific attribution of mitochondrial dysfunction is established by the spatial transcriptomics and deconvolution data (Figures 6, 7), which localize transcriptional suppression specifically to CA1 pyramidal neurons. CA1 showed pronounced downregulation of mitochondrial and bioenergetic gene programs (Figure 6L); by deconvolution, this is driven specifically by CA1.ProS pyramidal neurons, while CA3 neurons and glial populations maintain relatively stable mitochondrial gene profiles.

These observations are consistent with impaired oxidative phosphorylation in CA1 synapses. Prior studies using synaptosomes from 4- to 9-months-old 5xFAD mice demonstrated early mitochondrial dysfunction, including reduced respiration (Wang et al., 2016). In human AD tissue, structural mitochondrial changes including altered ER contacts have been documented (Area-Gomez et al., 2012). Cristae loss and structural abnormalities have long been implicated in neurodegenerative disease pathogenesis (Rhein et al., 2009; Ikon and Ryan, 2017).

### 4.4 Possible mechanisms underlying cristae disruption

Several converging mechanisms may contribute to, but have not been directly tested in this study as causes of the observed ultrastructural damage. These include: alterations in mitochondrial dynamics or cristae-organizing complexes (Li et al., 2004; Porcellotti et al., 2015); impaired mitophagy allowing damaged mitochondria to accumulate (Du et al., 2008; Zhang et al., 2016); direct Aβ perturbation of mitochondrial membrane curvature before plaque deposition (Rodrigues et al., 2001; Khalifat et al., 2012); mitochondrial permeability transition pore opening from calcium dysregulation (Rhein et al., 2009); and oxidative damage from impaired electron transport (Ikon and Ryan, 2017; Cole et al., 2010).

### 4.5 Spatially heterogeneous transcriptomic programs

CA1 showed suppression of mitochondrial/energetic pathways; DG showed transcriptomic signatures consistent with upregulation of interferon alpha and gamma response pathways alongside dysregulation of mitochondrial distribution and ion channel-related gene sets (Figure 6). Immunohistochemical quantification of Iba1+ microglial morphology and GFAP+ astrocyte reactivity would be required to determine whether this DG transcriptomic signature reflects active microglial or astrocyte neuroinflammation at this age. The DG interferon signature is noteworthy in light of recent studies implicating type I/II interferon signaling in synapse loss and microglial activation in amyloid-bearing models (Rhein et al., 2009; Minter et al., 2016; Roy et al., 2020, 2022). The spatial segregation of metabolic suppression (CA1) and interferon pathway upregulation (DG) is consistent with a model in which bioenergetic failure at synapses co-occurs with innate immune pathway engagement, though the relationship between these observations and active neuroinflammation requires direct investigation. We also observed genotype-dependent lipidomic changes in hippocampal subregions, consistent with early metabolic and membrane remodeling (Supplementary Figure 3).

### 4.6 Dendritic changes

Reductions in dendritic complexity in CA1 and dendritic length in DG were observed at 1 month. Declines in dendritic extent are characteristic of AD (Petrides et al., 2016; Coleman and Flood, 1987) and overexpression of Aβ in rodents is associated with decreased dendritic complexity (Golovyashkina et al., 2015; Wu et al., 2010). Non-ionotropic NMDA receptor signaling triggered by Aβ (Kessels et al., 2013) is associated with spine shrinkage via NOS1AP, nNOS, and cofilin (Stein et al., 2020). Increased spine density in DG granule cells of 5xFAD Hets contrasts with spine loss reports elsewhere and may reflect a transitional state involving loss of stable spines and gain of dynamic spines (Ortiz-Sanz et al., 2020), or may be related to the reduced dendritic length in the DG.

### 4.7 CA1 selective vulnerability

CA1 was more affected than CA3 or DG in both loss of dendritic complexity and transcriptomic changes. The CA1 region is more vulnerable to inflammation, hypoglycemia, ischemia, and excitotoxicity (Lahtinen et al., 2001; Kristensen et al., 2001; Bramlett et al., 1999; Alkadhi, 2019), and CA1 pathology occurs early in AD (Scheff et al., 2007; Braak and Braak, 1997; Kerchner et al., 2010, 2012; West et al., 1994). Our deconvolution analysis provides a transcriptomic basis for this: at 1 month, CA1.ProS neurons – but not CA3 neurons or DG granule cells – show a profound downregulation of mitochondrial respiratory machinery (Figure 7). Lower glutamate transporter expression (Zhang et al., 2011), higher GluN2B receptor density (Coultrap et al., 2005), and reduced calbindin-mediated calcium buffering (Sloviter, 1989) all are consistent with greater calcium-related vulnerability in CA1 compared to DG.

### 4.8 Linking mitochondrial findings to structural phenotypes

The correlation associations of structural phenotypes – reduced dendritic complexity in CA1, reduced dendritic length but increased spine density in DG, and sparing of CA3 – are consistent with the molecular signatures in the respective subregions. The spatial segregation suggests that different hippocampal subfields may engage different responses – energetic compensation or immune-driven remodeling – depending on local metabolic load, synaptic activity, Aβ exposure, and calcium handling.

### 4.9 Impacts on the animal

The behavioral and systemic consequences of the 1-month hippocampal phenotype described here were not directly investigated in this study, but published observations raise testable hypotheses. There is one group that reports spatial memory deficits as early as 1 month in 5xFAD Hets (Tang et al., 2016), and we have reported that both male and female 5xFAD Hets show sleep fragmentation prior to plaque deposition (Kim et al., 2025). Chronic sleep deprivation has been associated with dendritic simplification (Brodin et al., 2025) and mitochondrial morphology changes in the cortex and hippocampus (Lu et al., 2021; Sarnataro, 2025). Future studies incorporating behavioral assessment within the same animals that receive cellular analyses are needed to establish any link.

### 4.10 Limitations

Several important caveats should be acknowledged. First, we did not directly measure synaptic ATP levels, mitochondrial membrane potential, calcium, or ROS production *in situ*. The link between cristae disruption and energetic failure therefore remains inferential. Second, we cannot distinguish among the proposed mechanisms for cristae destruction, receptor hyperactivity, and dendritic simplification; detailed mechanistic experiments will be needed. Third, the study is correlational and descriptive; no pharmacological or genetic intervention was performed to establish causal relationships. Fourth, the interferon-related transcriptomic signatures in DG were not validated by IHC for microglial or astrocyte activation, representing an important gap. Fifth, electrophysiology experiments involved exclusively female mice; whether the AMPAR and GluN2B hyperexcitability phenotype is equivalent in males at 1 month has not been determined. Sixth, the morphometric and transcriptomic analyses used combined sexes; sex-stratified analyses are warranted in future studies. Seventh, mitochondrial size was quantified as 2D cross-sectional area from single-plane STEM images; volumetric measurements by serial section EM or FIB-SEM would provide more definitive size data in future studies.

## 5 Conclusion

In summary, our integrated analyses reveal that glutamate receptor hyperactivity, synaptic mitochondrial damage, suppressed bioenergetic gene programs, and region-specific transcriptomic changes consistent with interferon pathway upregulation are among the earliest detectable perturbations in the 5xFAD hippocampus – occurring well before overt amyloid pathology or neuronal loss. Critically, the mitochondrial transcriptomic suppression is restricted to CA1 pyramidal neurons, providing a cell-type-specific signature of bioenergetic vulnerability at 1 month of age. These findings identify a pre-plaque phenotypic constellation that motivates future mechanistic and interventional studies. Targeting mitochondrial preservation and immune modulation may represent promising early intervention strategies in both familial and sporadic Alzheimer’s disease.

## Data Availability

The datasets presented in this study can be found in online repositories. The names of the repository/repositories and accession number(s) can be found below: https://metaspace2020.org/datasets?q=2026-02-03_18h41m55s, 2026-02-03_18h41m55s https://doi.org/10.7267/5999nc87c, 5999nc87c.

## Footnotes

1
https://www.jax.org/Protocol?stockNumber=008730&protocolID=46804

## References

[B1] Alberdi E. Sánchez-Gómez M. V. Cavaliere F. Pérez-Samartín A. Zugaza J. L. Trullas R. et al. (2010). Amyloid beta oligomers induce Ca2+ dysregulation and neuronal death through activation of ionotropic glutamate receptors. *Cell Calcium* 47 264–272. 10.1016/j.ceca.2009.12.010 20061018

[B2] Alessandri B. Bättig K. Welzl H. (1989). Effects of ketamine on tunnel maze and water maze performance in the rat. *Behav. Neural. Biol.* 52 194–212. 10.1016/s0163-1047(89)90313-0 2552977

[B3] Alkadhi K. A. (2019). Cellular and molecular differences between area CA1 and the dentate gyrus of the hippocampus. *Mol. Neurobiol.* 56 6566–6580. 10.1007/s12035-019-1541-2 30874972

[B4] Alzheimer’s Association. (2024). 2024 Alzheimer’s disease facts and figures. *Alzheimers Dement.* 20, 3708–3821. 10.1002/alz.13809 38689398 PMC11095490

[B5] Area-Gomez E. Del Carmen Lara Castillo M. Tambini M. D. Guardia-Laguarta C. de Groof A. J. Madra M. et al. (2012). Upregulated function of mitochondria-associated ER membranes in Alzheimer disease. *EMBO J.* 31 4106–4123. 10.1038/emboj.2012.202 22892566 PMC3492725

[B6] Armstrong R. A. (2006). Plaques and tangles and the pathogenesis of Alzheimer’s disease. *Folia Neuropathol.* 44 1–11. 16565925

[B7] Ashburner M. Ball C. A. Blake J. A. Botstein D. Butler H. Cherry J. M. et al. (2000). Gene ontology: Tool for the unification of biology. the gene ontology consortium. *Nat. Genet.* 25 25–29. 10.1038/75556 10802651 PMC3037419

[B8] Auffret A. Gautheron V. Mattson M. P. Mariani J. Rovira C. (2010). Progressive age-related impairment of the late long-term potentiation in Alzheimer’s disease presenilin-1 mutant knock-in mice. *J. Alzheimers Dis.* 19 1021–1033. 10.3233/JAD-2010-1302 20157256 PMC2891870

[B9] Auffret A. Gautheron V. Repici M. Kraftsik R. Mount H. T. Mariani J. et al. (2009). Age-dependent impairment of spine morphology and synaptic plasticity in hippocampal CA1 neurons of a presenilin 1 transgenic mouse model of Alzheimer’s disease. *J. Neurosci.* 29 10144–10152. 10.1523/JNEUROSCI.1856-09.2009 19675248 PMC6664983

[B10] Bader A. S. Gnädig M. U. Fricke M. Büschgens L. Berger L. J. Klafki H. W. et al. (2023). Brain region-specific differences in Amyloid-β plaque composition in 5XFAD mice. *Life* 13:1053. 10.3390/life13041053 37109582 PMC10145597

[B11] Bateman R. J. Xiong C. Benzinger T. L. Fagan A. M. Goate A. Fox N. C. et al. (2012). Clinical and biomarker changes in dominantly inherited Alzheimer’s disease. *N. Engl. J. Med.* 367 795–804. 10.1056/NEJMoa1202753 22784036 PMC3474597

[B12] Billings L. M. Oddo S. Green K. N. McGaugh J. L. LaFerla F. M. (2005). Intraneuronal Abeta causes the onset of early Alzheimer’s disease-related cognitive deficits in transgenic mice. *Neuron* 45 675–688. 10.1016/j.neuron.2005.01.040 15748844

[B13] Bittner T. Burgold S. Dorostkar M. M. Fuhrmann M. Wegenast-Braun B. M. Schmidt B. et al. (2012). Amyloid plaque formation precedes dendritic spine loss. *Acta Neuropathol.* 124 797–807. 10.1007/s00401-012-1047-8 22993126 PMC3508278

[B14] Blazquez-Llorca L. Valero-Freitag S. Rodrigues E. F. Merchán-Pérez Á Rodríguez J. R. Dorostkar M. M. et al. (2017). High plasticity of axonal pathology in Alzheimer’s disease mouse models. *Acta Neuropathol. Commun.* 5:14. 10.1186/s40478-017-0415-y 28173876 PMC5296955

[B15] Braak E. Braak H. (1997). Alzheimer’s disease: Transiently developing dendritic changes in pyramidal cells of sector CA1 of the Ammon’s horn. *Acta Neuropathol.* 93 323–325. 10.1007/s004010050622 9113196

[B16] Bramlett H. M. Green E. J. Dietrich W. D. (1999). Exacerbation of cortical and hippocampal CA1 damage due to posttraumatic hypoxia following moderate fluid-percussion brain injury in rats. *J. Neurosurg.* 91 653–659. 10.3171/jns.1999.91.4.0653 10507388

[B17] Brodin A. T. S. Liesecke F. Spielbauer J. Karlsson T. E. (2025). Sleep deprivation and dendritic architecture: A systematic review and meta-analysis. *Sleep* 48:zsaf146. 10.1093/sleep/zsaf146 40462346 PMC12417018

[B18] Buccellato F. R. D’Anca M. Tartaglia G. M. Del Fabbro M. Scarpini E. Galimberti D. (2023). Treatment of Alzheimer’s disease: Beyond symptomatic therapies. *Int. J. Mol. Sci.* 24:13900. 10.3390/ijms241813900 37762203 PMC10531090

[B19] Busche M. A. Chen X. Henning H. A. Reichwald J. Staufenbiel M. Sakmann B. et al. (2012). Critical role of soluble amyloid-β for early hippocampal hyperactivity in a mouse model of Alzheimer’s disease. *Proc. Natl. Acad. Sci. U. S. A.* 109 8740–8745. 10.1073/pnas.1206171109 22592800 PMC3365221

[B20] Butelman E. R. (1989). A novel NMDA antagonist, MK-801, impairs performance in a hippocampal-dependent spatial learning task. *Pharmacol. Biochem. Behav.* 34 13–16. 10.1016/0091-3057(89)90345-6 2696982

[B21] Choi D. W. (1992). Excitotoxic cell death. *J. Neurobiol.* 23 1261–1276. 10.1002/neu.480230915 1361523

[B22] Citron M. Eckman C. B. Diehl T. S. Corcoran C. Ostaszewski B. L. Xia W. et al. (1998). Additive effects of PS1 and APP mutations on secretion of the 42-residue amyloid beta-protein. *Neurobiol. Dis.* 5 107–116. 10.1006/nbdi.1998.0183 9746908

[B23] Cole N. B. Daniels M. P. Levine R. L. Kim G. (2010). Oxidative stress causes reversible changes in mitochondrial permeability and structure. *Exp. Gerontol.* 45 596–602. 10.1016/j.exger.2010.01.016 20096768 PMC2879436

[B24] Coleman P. D. Flood D. G. (1987). Neuron numbers and dendritic extent in normal aging and Alzheimer’s disease. *Neurobiol. Aging* 8 521–545. 10.1016/0197-4580(87)90127-8 3323927

[B25] Costa R. O. Lacor P. N. Ferreira I. L. Resende R. Auberson Y. P. Klein W. L. et al. (2012). Endoplasmic reticulum stress occurs downstream of GluN2B subunit of N-methyl-d-aspartate receptor in mature hippocampal cultures treated with amyloid-β oligomers. *Aging Cell* 11 823–833. 10.1111/j.1474-9726.2012.00848.x 22708890

[B26] Coultrap S. J. Nixon K. M. Alvestad R. M. Valenzuela C. F. Browning M. D. (2005). Differential expression of NMDA receptor subunits and splice variants among the CA1, CA3 and dentate gyrus of the adult rat. *Brain Res. Mol. Brain Res.* 135 104–111. 10.1016/j.molbrainres.2004.12.005 15857673

[B27] Dewachter I. Filipkowski R. K. Priller C. Ris L. Neyton J. Croes S. et al. (2009). Deregulation of NMDA-receptor function and down-stream signaling in APP[V717I] transgenic mice. *Neurobiol. Aging* 30 241–256. 10.1016/j.neurobiolaging.2007.06.011 17673336

[B28] Dewachter I. Ris L. Croes S. Borghgraef P. Devijver H. Voets T. et al. (2008). Modulation of synaptic plasticity and Tau phosphorylation by wild-type and mutant presenilin1. *Neurobiol. Aging* 29 639–652. 10.1016/j.neurobiolaging.2006.11.019 17222948

[B29] Dickerson B. C. Salat D. H. Greve D. N. Chua E. F. Rand-Giovannetti E. Rentz D. M. et al. (2005). Increased hippocampal activation in mild cognitive impairment compared to normal aging and AD. *Neurology* 65 404–411. 10.1212/01.wnl.0000171450.97464.49 16087905 PMC4335677

[B30] Du H. Guo L. Fang F. Chen D. Sosunov A. A. McKhann G. M. et al. (2008). Cyclophilin D deficiency attenuates mitochondrial and neuronal perturbation and ameliorates learning and memory in Alzheimer’s disease. *Nat. Med.* 14 1097–1105. 10.1038/nm.1868 18806802 PMC2789841

[B31] Eckman C. B. Mehta N. D. Crook R. Perez-tur J. Prihar G. Pfeiffer E. et al. (1997). A new pathogenic mutation in the APP gene (I716V) increases the relative proportion of A beta 42(43). *Hum. Mol. Genet.* 6 2087–2089. 10.1093/hmg/6.12.2087 9328472

[B32] Eimer W. A. Vassar R. (2013). Neuron loss in the 5XFAD mouse model of Alzheimer’s disease correlates with intraneuronal Aβ42 accumulation and Caspase-3 activation. *Mol. Neurodegener.* 8:2. 10.1186/1750-1326-8-2 23316765 PMC3552866

[B33] Esler W. P. Wolfe M. S. (2001). A portrait of Alzheimer secretases–new features and familiar faces. *Science* 293 1449–1454. 10.1126/science.1064638 11520976

[B34] Ferreira I. L. Ferreiro E. Schmidt J. Cardoso J. M. Pereira C. M. Carvalho A. L. et al. (2015). Aβ and NMDAR activation cause mitochondrial dysfunction involving ER calcium release. *Neurobiol. Aging* 36 680–692. 10.1016/j.neurobiolaging.2014.09.006 25442114

[B35] Foster C. M. Kennedy K. M. Horn M. M. Hoagey D. A. Rodrigue K. M. (2018). Both hyper- and hypo-activation to cognitive challenge are associated with increased beta-amyloid deposition in healthy aging: A nonlinear effect. *Neuroimage* 166 285–292. 10.1016/j.neuroimage.2017.10.068 29108941 PMC5747976

[B36] Gail Canter R. Huang W. C. Choi H. Wang J. Ashley Watson L. Yao C. G. et al. (2019). 3D mapping reveals network-specific amyloid progression and subcortical susceptibility in mice. *Commun. Biol.* 2:360. 10.1038/s42003-019-0599-8 31602409 PMC6778135

[B37] Gene Ontology C. Aleksander S. A. Balhoff J. Carbon S. Cherry J. M. Drabkin H. J. et al. (2023). The gene ontology knowledgebase in 2023. *Genetics* 224:iyad031. 10.1093/genetics/iyad031 36866529 PMC10158837

[B38] Gillespie M. Jassal B. Stephan R. Milacic M. Rothfels K. Senff-Ribeiro A. et al. (2022). The reactome pathway knowledgebase 2022. *Nucleic Acids Res.* 50 D687–D692. 10.1093/nar/gkab1028 34788843 PMC8689983

[B39] Golovyashkina N. Penazzi L. Ballatore C. Smith A. B. Bakota L. Brandt R. (2015). Region-specific dendritic simplification induced by Aβ, mediated by tau via dysregulation of microtubule dynamics: A mechanistic distinct event from other neurodegenerative processes. *Mol. Neurodegener.* 10:60. 10.1186/s13024-015-0049-0 26541821 PMC4634596

[B40] Grutzendler J. Helmin K. Tsai J. Gan W. B. (2007). Various dendritic abnormalities are associated with fibrillar amyloid deposits in Alzheimer’s disease. *Ann. N. Y. Acad. Sci.* 1097 30–39. 10.1196/annals.1379.003 17413007

[B41] Heale V. Harley C. (1990). MK-801 and AP5 impair acquisition, but not retention, of the Morris milk maze. *Pharmacol. Biochem. Behav.* 36 145–149. 10.1016/0091-3057(90)90140-d 1971949

[B42] Hermann D. Both M. Ebert U. Gross G. Schoemaker H. Draguhn A. et al. (2009). Synaptic transmission is impaired prior to plaque formation in amyloid precursor protein-overexpressing mice without altering behaviorally-correlated sharp wave-ripple complexes. *Neuroscience* 162 1081–1090. 10.1016/j.neuroscience.2009.05.044 19477243

[B43] Hong S. Beja-Glasser V. F. Nfonoyim B. M. Frouin A. Li S. Ramakrishnan S. et al. (2016). Complement and microglia mediate early synapse loss in Alzheimer mouse models. *Science* 352 712–716. 10.1126/science.aad8373 27033548 PMC5094372

[B44] Hunter J. D. (2007). Matplotlib: A 2D graphics environment. *Comp. Sci. Eng.* 9 90–95. 10.1109/MCSE.2007.55

[B45] Ikon N. Ryan R. O. (2017). Cardiolipin and mitochondrial cristae organization. *Biochim Biophys. Acta Biomembr.* 1859 1156–1163. 10.1016/j.bbamem.2017.03.013 28336315 PMC5426559

[B46] Jack C. R. Knopman D. S. Jagust W. J. Petersen R. C. Weiner M. W. Aisen P. S. et al. (2013). Tracking pathophysiological processes in Alzheimer’s disease: An updated hypothetical model of dynamic biomarkers. *Lancet Neurol.* 12 207–216. 10.1016/S1474-4422(12)70291-0 23332364 PMC3622225

[B47] Jankowsky J. L. Slunt H. H. Gonzales V. Jenkins N. A. Copeland N. G. Borchelt D. R. (2004). APP processing and amyloid deposition in mice haplo-insufficient for presenilin 1. *Neurobiol. Aging* 25 885–892. 10.1016/j.neurobiolaging.2003.09.008 15212842

[B48] Jawhar S. Trawicka A. Jenneckens C. Bayer T. A. Wirths O. (2012). Motor deficits, neuron loss, and reduced anxiety coinciding with axonal degeneration and intraneuronal Aβ aggregation in the 5XFAD mouse model of Alzheimer’s disease. *Neurobiol. Aging* 33 196.e29–40. 10.1016/j.neurobiolaging.2010.05.027 20619937

[B49] Karl T. Bhatia S. Cheng D. Kim W. S. Garner B. (2012). Cognitive phenotyping of amyloid precursor protein transgenic J20 mice. *Behav. Brain Res.* 228 392–397. 10.1016/j.bbr.2011.12.021 22197298

[B50] Kerchner G. A. Deutsch G. K. Zeineh M. Dougherty R. F. Saranathan M. Rutt B. K. (2012). Hippocampal CA1 apical neuropil atrophy and memory performance in Alzheimer’s disease. *Neuroimage* 63 194–202. 10.1016/j.neuroimage.2012.06.048 22766164 PMC3677969

[B51] Kerchner G. A. Hess C. P. Hammond-Rosenbluth K. E. Xu D. Rabinovici G. D. Kelley D. A. et al. (2010). Hippocampal CA1 apical neuropil atrophy in mild Alzheimer disease visualized with 7-T MRI. *Neurology* 75 1381–1387. 10.1212/WNL.0b013e3181f736a1 20938031 PMC3013485

[B52] Kessels H. W. Nabavi S. Malinow R. (2013). Metabotropic NMDA receptor function is required for β-amyloid-induced synaptic depression. *Proc. Natl. Acad. Sci. U. S. A.* 110 4033–4038. 10.1073/pnas.1219605110 23431156 PMC3593880

[B53] Khalifat N. Puff N. Dliaa M. Angelova M. I. (2012). Amyloid-β and the failure to form mitochondrial cristae: A biomimetic study involving artificial membranes. *J. Alzheimers Dis.* 28 33–48. 10.3233/JAD-2011-110389 21987591

[B54] Kim K. H. Moon M. Yu S. B. Mook-Jung I. Kim J. I. (2012). RNA-Seq analysis of frontal cortex and cerebellum from 5XFAD mice at early stage of disease pathology. *J. Alzheimers Dis.* 29 793–808. 10.3233/JAD-2012-111793 22507954

[B55] Kim K. J. Villegas A. L. Kelley A. R. Labut E. M. Hagen T. M. Magnusson K. R. et al. (2025). Sex differences in sleep fragmentation in 5xFAD mice. *Neuroscience* 589 118–127. 10.1016/j.neuroscience.2025.10.035 41138967

[B56] Korotkevich G. Sukhov V. Sergushichev A. (2019). Fast gene set enrichment analysis. *bioRxiv [Preprint].* 10.1101/060012

[B57] Krafft G. A. Jerecic J. Siemers E. Cline E. N. (2022). ACU193: An immunotherapeutic poised to test the amyloid β oligomer hypothesis of Alzheimer’s disease. *Front. Neurosci.* 16:848215. 10.3389/fnins.2022.848215 35557606 PMC9088393

[B58] Kristensen B. W. Noraberg J. Zimmer J. (2001). Comparison of excitotoxic profiles of ATPA, AMPA, KA and NMDA in organotypic hippocampal slice cultures. *Brain Res.* 917 21–44. 10.1016/s0006-8993(01)02900-6 11602227

[B59] Lahtinen H. Autere A. M. Paalasmaa P. Lauri S. E. Kaila K. (2001). Post-insult activity is a major cause of delayed neuronal death in organotypic hippocampal slices exposed to glutamate. *Neuroscience* 105 131–137. 10.1016/s0306-4522(01)00168-3 11483307

[B60] Li S. Hong S. Shepardson N. E. Walsh D. M. Shankar G. M. Selkoe D. (2009). Soluble oligomers of amyloid Beta protein facilitate hippocampal long-term depression by disrupting neuronal glutamate uptake. *Neuron* 62 788–801. 10.1016/j.neuron.2009.05.012 19555648 PMC2702854

[B61] Li S. Jin M. Koeglsperger T. Shepardson N. E. Shankar G. M. Selkoe D. J. (2011). Soluble Aβ oligomers inhibit long-term potentiation through a mechanism involving excessive activation of extrasynaptic NR2B-containing NMDA receptors. *J. Neurosci.* 31 6627–6638. 10.1523/JNEUROSCI.0203-11.2011 21543591 PMC3100898

[B62] Li Z. Okamoto K. Hayashi Y. Sheng M. (2004). The importance of dendritic mitochondria in the morphogenesis and plasticity of spines and synapses. *Cell* 119 873–887. 10.1016/j.cell.2004.11.003 15607982

[B63] Liberzon A. Birger C. Thorvaldsdóttir H. Ghandi M. Mesirov J. P. Tamayo P. (2015). The Molecular Signatures Database (MSigDB) hallmark gene set collection. *Cell Syst.* 1 417–425. 10.1016/j.cels.2015.12.004 26771021 PMC4707969

[B64] Liberzon A. Subramanian A. Pinchback R. Thorvaldsdóttir H. Tamayo P. Mesirov J. P. (2011). Molecular signatures database (MSigDB) 3.0. *Bioinformatics* 27 1739–1740. 10.1093/bioinformatics/btr260 21546393 PMC3106198

[B65] Longair M. H. Baker D. A. Armstrong J. D. (2011). Simple Neurite Tracer: Open source software for reconstruction, visualization and analysis of neuronal processes. *Bioinformatics* 27 2453–2454. 10.1093/bioinformatics/btr390 21727141

[B66] Lu Z. Hu Y. Wang Y. Zhang T. Long J. Liu J. (2021). Topological reorganizations of mitochondria isolated from rat brain after 72 hours of paradoxical sleep deprivation, revealed by electron cryo-tomography. *Am. J. Physiol. Cell Physiol.* 321 C17–C25. 10.1152/ajpcell.00077.2021 33979213

[B67] Ma T. Cheng Q. Chen C. Luo Z. Feng D. (2020). Excessive activation of NMDA receptors in the pathogenesis of multiple peripheral organs via mitochondrial dysfunction, oxidative stress, and inflammation. *SN Comprehens. Clin. Med.* 2 551–569. 10.1007/s42399-020-00298-w

[B68] Magnusson K. R. (1998). The aging of the NMDA receptor complex. *Front. Biosci.* 3:e70–e80. 10.2741/a368 9576682

[B69] Magnusson K. R. Cotman C. W. (1993). Age-related changes in excitatory amino acid receptors in two mouse strains. *Neurobiol. Aging* 14 197–206. 10.1016/0197-4580(93)90001-r 8391661

[B70] Masters C. L. Bateman R. Blennow K. Rowe C. C. Sperling R. A. Cummings J. L. (2015). Alzheimer’s disease. *Nat. Rev. Dis Primers* 1:15056. 10.1038/nrdp.2015.56 27188934

[B71] McInnes L. Healy J. Melville J. (2020). UMAP: Uniform manifold approximation and projection for dimension reduction. *arXiv [Preprint].* 10.48550/arXiv.1802.03426

[B72] Mertaş B. Boşgelmez I. İ (2025). The role of genetic, environmental, and dietary factors in Alzheimer’s disease: A narrative review. *Int. J. Mol. Sci.* 26:1222. 10.3390/ijms26031222 39940989 PMC11818526

[B73] Minter M. R. Moore Z. Zhang M. Brody K. M. Jones N. C. Shultz S. R. et al. (2016). Deletion of the type-1 interferon receptor in APPSWE/PS1ΔE9 mice preserves cognitive function and alters glial phenotype. *Acta Neuropathol. Commun.* 4:72. 10.1186/s40478-016-0341-4 27400725 PMC4940712

[B74] Mirra S. S. Heyman A. McKeel D. Sumi S. M. Crain B. J. Brownlee L. M. et al. (1991). The Consortium to Establish a Registry for Alzheimer’s Disease (CERAD). Part II. Standardization of the neuropathologic assessment of Alzheimer’s disease. *Neurology* 41 479–486. 10.1212/wnl.41.4.479 2011243

[B75] Mondadori C. Weiskrantz L. Buerki H. Petschke F. Fagg G. E. (1989). NMDA receptor antagonists can enhance or impair learning performance in animals. *Exp. Brain Res.* 75 449–456. 10.1007/BF00249896 2545467

[B76] Mondragón-Rodríguez S. Basurto-Islas G. Lee H. G. Perry G. Zhu X. Castellani R. J. et al. (2010). Causes versus effects: The increasing complexities of Alzheimer’s disease pathogenesis. *Exp. Rev. Neurother* 10 683–691. 10.1586/ern.10.27 20420489 PMC2922904

[B77] Mootha V. K. Lindgren C. M. Eriksson K. F. Subramanian A. Sihag S. Lehar J. et al. (2003). PGC-1alpha-responsive genes involved in oxidative phosphorylation are coordinately downregulated in human diabetes. *Nat. Genet.* 34 267–273. 10.1038/ng1180 12808457

[B78] Mormino E. C. Brandel M. G. Madison C. M. Marks S. Baker S. L. Jagust W. J. (2012). Aβ Deposition in aging is associated with increases in brain activation during successful memory encoding. *Cereb. Cortex* 22 1813–1823. 10.1093/cercor/bhr255 21945849 PMC3388896

[B79] Morris R. G. M. Anderson E. Lynch G. S. Baudry M. (1986). Selective impairment of learning and blockade of long-term potentiation by an N-methyl-D-aspartate receptor antagonist. AP5. *Nature* 319 774–776. 10.1038/319774a0 2869411

[B80] Mosconi L. Glucose metabolism in normal aging and Alzheimer’s disease: Methodological and physiological considerations for PET studies. *Clin. Transl. Imag.* 2013:10.1007/s40336-013-0026-y. 10.1007/s40336-013-0026-y. 24409422 PMC3881550

[B81] Mucke L. Masliah E. Yu G. Q. Mallory M. Rockenstein E. M. Tatsuno G. et al. (2000). High-level neuronal expression of abeta 1-42 in wild-type human amyloid protein precursor transgenic mice: Synaptotoxicity without plaque formation. *J. Neurosci.* 20 4050–4058. 10.1523/JNEUROSCI.20-11-04050.2000 10818140 PMC6772621

[B82] Mustaly-Kalimi S. Gallegos W. Steinbrenner D. Gupta S. Houcek A. J. Bennett D. A. et al. (2025). Mitochondrial dysfunction mediated by ER-calcium dysregulation in neurons derived from Alzheimer’s disease patients. *Acta Neuropathol. Commun.* 13:165. 10.1186/s40478-025-02023-x 40721842 PMC12305934

[B83] National Institute on Aging (2016). *Alzheimer’s Disease Fact Sheet.* Bethesda, MD: National Institute on Aging.

[B84] Oakley H. Cole S. L. Logan S. Maus E. Shao P. Craft J. et al. (2006). Intraneuronal beta-amyloid aggregates, neurodegeneration, and neuron loss in transgenic mice with five familial Alzheimer’s disease mutations: Potential factors in amyloid plaque formation. *J. Neurosci.* 26 10129–10140. 10.1523/JNEUROSCI.1202-06.2006 17021169 PMC6674618

[B85] Oddo S. Caccamo A. Shepherd J. D. Murphy M. P. Golde T. E. Kayed R. et al. (2003). Triple-transgenic model of Alzheimer’s disease with plaques and tangles: Intracellular Abeta and synaptic dysfunction. *Neuron* 39 409–421. 10.1016/s0896-6273(03)00434-3 12895417

[B86] Ortiz-Sanz C. Gaminde-Blasco A. Valero J. Bakota L. Brandt R. Zugaza J. L. et al. (2020). Early effects of Aβ oligomers on dendritic spine dynamics and arborization in hippocampal neurons. *Front. Synaptic Neurosci.* 12:2. 10.3389/fnsyn.2020.00002 32116638 PMC7029715

[B87] Perez-Cruz C. Nolte M. W. Van Gaalen M. M. Rustay N. R. Termont A. Tanghe A. et al. (2011). Reduced spine density in specific regions of CA1 pyramidal neurons in two transgenic mouse models of Alzheimer’s disease. *J. Neurosci.* 31 3926–3934. 10.1523/JNEUROSCI.6142-10.2011 21389247 PMC6622797

[B88] Petrides F. E. Mavroudis I. A. Dados D. Manani M. G. Chatzinikolaou F. G. Spilioti M. et al. (2016). Dendritic alterations of the pyramidal cell of reil’s insula in Alzheimer’s disease. *J. Neurol. Stroke* 4:00154. 10.15406/jnsk.2016.04.00154

[B89] Porcellotti S. Fanelli F. Fracassi A. Sepe S. Cecconi F. Bernardi C. et al. (2015). Oxidative stress during the progression of β-amyloid pathology in the neocortex of the Tg2576 mouse model of Alzheimer’s disease. *Oxid. Med. Cell Longev.* 2015:967203. 10.1155/2015/967203 25973140 PMC4418010

[B90] Preuss C. Pandey R. Piazza E. Fine A. Uyar A. Perumal T. et al. (2020). A novel systems biology approach to evaluate mouse models of late-onset Alzheimer’s disease. *Mol. Neurodegener.* 15:67. 10.1186/s13024-020-00412-5 33172468 PMC7656729

[B91] Ray A. Loghinov I. Ravindranath V. Barth A. L. (2024). Early hippocampal hyperexcitability and synaptic reorganization in mouse models of amyloidosis. *iScience* 27:110629. 10.1016/j.isci.2024.110629 39262788 PMC11388185

[B92] Rhein V. Baysang G. Rao S. Meier F. Bonert A. Müller-Spahn F. et al. (2009). Amyloid-beta leads to impaired cellular respiration, energy production and mitochondrial electron chain complex activities in human neuroblastoma cells. *Cell Mol. Neurobiol.* 29 1063–1071. 10.1007/s10571-009-9398-y 19350381 PMC11506282

[B93] Richard B. C. Kurdakova A. Baches S. Bayer T. A. Weggen S. Wirths O. (2015). Gene dosage dependent aggravation of the neurological phenotype in the 5XFAD mouse model of Alzheimer’s disease. *J. Alzheimers Dis.* 45 1223–1236. 10.3233/JAD-143120 25697701

[B94] Rodrigues C. M. Solá S. Brito M. A. Brondino C. D. Brites D. Moura J. J. (2001). Amyloid beta-peptide disrupts mitochondrial membrane lipid and protein structure: Protective role of tauroursodeoxycholate. *Biochem. Biophys. Res. Commun.* 281 468–474. 10.1006/bbrc.2001.4370 11181071

[B95] Roy E. R. Chiu G. Li S. Propson N. E. Kanchi R. Wang B. et al. (2022). Concerted type I interferon signaling in microglia and neural cells promotes memory impairment associated with amyloid β plaques. *Immunity* 55 879–894.e6. 10.1016/j.immuni.2022.03.018 35443157 PMC9109419

[B96] Roy E. R. Wang B. Wan Y. W. Chiu G. Cole A. Yin Z. et al. (2020). Type I interferon response drives neuroinflammation and synapse loss in Alzheimer disease. *J. Clin. Invest.* 130 1912–1930. 10.1172/JCI133737 31917687 PMC7108898

[B97] Ryan K. C. Ashkavand Z. Norman K. R. (2020). The role of mitochondrial calcium homeostasis in Alzheimer’s and related diseases. *Int. J. Mol. Sci.* 21:9153. 10.3390/ijms21239153 33271784 PMC7730848

[B98] Sarnataro R. (2025). Neurobiology of mitochondrial dynamics in sleep. *J. Physiol.* 603 6747–6762. 10.1113/JP288054 40846498 PMC12645550

[B99] Savonenko A. Xu G. M. Melnikova T. Morton J. L. Gonzales V. Wong M. P. et al. (2005). Episodic-like memory deficits in the APPswe/PS1dE9 mouse model of Alzheimer’s disease: Relationships to beta-amyloid deposition and neurotransmitter abnormalities. *Neurobiol. Dis.* 18 602–617. 10.1016/j.nbd.2004.10.022 15755686

[B100] Scheff S. W. Price D. A. Schmitt F. A. DeKosky S. T. Mufson E. J. (2007). Synaptic alterations in CA1 in mild Alzheimer disease and mild cognitive impairment. *Neurology* 68 1501–1508. 10.1212/01.wnl.0000260698.46517.8f 17470753

[B101] Schindelin J. Arganda-Carreras I. Frise E. Kaynig V. Longair M. Pietzsch T. et al. (2012). Fiji: An open-source platform for biological-image analysis. *Nat. Methods* 9 676–682. 10.1038/nmeth.2019 22743772 PMC3855844

[B102] Schneider I. Reverse D. Dewachter I. Ris L. Caluwaerts N. Kuiperi C. et al. (2001). Mutant presenilins disturb neuronal calcium homeostasis in the brain of transgenic mice, decreasing the threshold for excitotoxicity and facilitating long-term potentiation. *J. Biol. Chem.* 276 11539–11544. 10.1074/jbc.M010977200 11278803

[B103] Selkoe D. J. Hardy J. (2016). The amyloid hypothesis of Alzheimer’s disease at 25 years. *EMBO Mol. Med.* 8 595–608. 10.15252/emmm.201606210 27025652 PMC4888851

[B104] Sheikh M. Khan S. J. Butt H. A. T. Zaidi S. A. T. Na V. (2023). From symptomatic treatment to disease modification: A turning point in Alzheimer’s disease management. *Cureus* 15:e47251. 10.7759/cureus.47251 38021811 PMC10655160

[B105] Shi Y. B. Tu T. Jiang J. Zhang Q. L. Ai J. Q. Pan A. et al. (2020). Early dendritic dystrophy in human brains with primary age-related tauopathy. *Front. Aging Neurosci.* 12:596894. 10.3389/fnagi.2020.596894 33364934 PMC7750631

[B106] Sloviter R. S. (1989). Calcium-binding protein (calbindin-D28k) and parvalbumin immunocytochemistry: Localization in the rat hippocampus with specific reference to the selective vulnerability of hippocampal neurons to seizure activity. *J. Comp. Neurol.* 280 183–196. 10.1002/cne.903080306 2925892

[B107] Stanika R. I. Pivovarova N. B. Brantner C. A. Watts C. A. Winters C. A. Andrews S. B. (2009). Coupling diverse routes of calcium entry to mitochondrial dysfunction and glutamate excitotoxicity. *Proc. Natl. Acad. Sci. U. S. A.* 106 9854–9859. 10.1073/pnas.0903546106 19482936 PMC2701040

[B108] Stein I. S. Park D. K. Flores J. C. Jahncke J. N. Zito K. (2020). Molecular mechanisms of non-ionotropic NMDA receptor signaling in dendritic spine shrinkage. *J. Neurosci.* 40 3741–3750. 10.1523/JNEUROSCI.0046-20.2020 32321746 PMC7204083

[B109] Subramanian A. Tamayo P. Mootha V. K. Mukherjee S. Ebert B. L. Gillette M. A. et al. (2005). Gene set enrichment analysis: A knowledge-based approach for interpreting genome-wide expression profiles. *Proc. Natl. Acad. Sci. U. S. A.* 102 15545–15550. 10.1073/pnas.0506580102 16199517 PMC1239896

[B110] Takami M. Nagashima Y. Sano Y. Ishihara S. Morishima-Kawashima M. Funamoto S. et al. (2009). gamma-Secretase: Successive tripeptide and tetrapeptide release from the transmembrane domain of beta-carboxyl terminal fragment. *J. Neurosci.* 29 13042–13052. 10.1523/JNEUROSCI.2362-09.2009 19828817 PMC6665297

[B111] Talantova M. Sanz-Blasco S. Zhang X. Xia P. Akhtar M. W. Okamoto S. et al. (2013). Aβ induces astrocytic glutamate release, extrasynaptic NMDA receptor activation, and synaptic loss. *Proc. Natl. Acad. Sci. U. S. A.* 110 E2518–E2527. 10.1073/pnas.1306832110 23776240 PMC3704025

[B112] Tang X. Wu D. Gu L. H. Nie B. B. Qi X. Y. Wang Y. J. et al. (2016). Spatial learning and memory impairments are associated with increased neuronal activity in 5XFAD mouse as measured by manganese-enhanced magnetic resonance imaging. *Oncotarget* 7 57556–57570. 10.18632/oncotarget.11353 27542275 PMC5295372

[B113] Tarazona S. Furió-Tarí P. Turrà D. Pietro A. D. Nueda M. J. Ferrer A. et al. (2015). Data quality aware analysis of differential expression in RNA-seq with NOISeq R/Bioc package. *Nucleic Acids Res.* 43:e140. 10.1093/nar/gkv711 26184878 PMC4666377

[B114] Tolar M. Hey J. Power A. Abushakra S. (2021). Neurotoxic soluble amyloid oligomers drive Alzheimer’s pathogenesis and represent a clinically validated target for slowing disease progression. *Int. J. Mol. Sci.* 22:6355. 10.3390/ijms22126355 34198582 PMC8231952

[B115] van der Maaten L. Hinton G. (2008). Visualizing data using t-SNE. *J. Mach. Learn. Res.* 9 2579–2605.

[B116] Walters G. C. Usachev Y. M. (2023). Mitochondrial calcium cycling in neuronal function and neurodegeneration. *Front. Cell Dev. Biol.* 11:1094356. 10.3389/fcell.2023.1094356 36760367 PMC9902777

[B117] Wang L. Guo L. Lu L. Sun H. Shao M. Beck S. J. et al. (2016). Synaptosomal mitochondrial dysfunction in 5xFAD mouse model of Alzheimer’s disease. *PLoS One* 11:e0150441. 10.1371/journal.pone.0150441 26942905 PMC4778903

[B118] Wang Y. Greig N. H. Yu Q. S. Mattson M. P. (2009). Presenilin-1 mutation impairs cholinergic modulation of synaptic plasticity and suppresses NMDA currents in hippocampus slices. *Neurobiol. Aging* 30 1061–1068. 10.1016/j.neurobiolaging.2007.10.009 18068871 PMC2717610

[B119] West M. J. Coleman P. D. Flood D. G. Troncoso J. C. (1994). Differences in the pattern of hippocampal neuronal loss in normal ageing and Alzheimer’s disease. *Lancet* 344 769–772. 10.1016/s0140-6736(94)92338-8 7916070

[B120] Wickham H. (2016). *ggplot2: Elegant Graphics for Data Analysis.* New York, NY: Springer-Verlag.

[B121] Wu D. Tang X. Gu L. H. Li X. L. Qi X. Y. Bai F. et al. (2018). LINGO-1 antibody ameliorates myelin impairment and spatial memory deficits in the early stage of 5XFAD mice. *CNS Neurosci. Ther.* 24 381–393. 10.1111/cns.12809 29427384 PMC6489849

[B122] Wu H. Y. Hudry E. Hashimoto T. Kuchibhotla K. Rozkalne A. Fan Z. et al. (2010). Amyloid beta induces the morphological neurodegenerative triad of spine loss, dendritic simplification, and neuritic dystrophies through calcineurin activation. *J. Neurosci.* 30 2636–2649. 10.1523/JNEUROSCI.4456-09.2010 20164348 PMC2841957

[B123] Wu L. Rosa-Neto P. Hsiung G. Y. Sadovnick A. D. Masellis M. Black S. E. et al. (2012). Early-onset familial Alzheimer’s disease (EOFAD). *Can. J. Neurol. Sci.* 39 436–445. 10.1017/s0317167100013949 22728850

[B124] Yao Z. van Velthoven C. T. J. Nguyen T. N. Goldy J. Sedeno-Cortes A. E. Baftizadeh F. et al. (2021). A taxonomy of transcriptomic cell types across the isocortex and hippocampal formation. *Cell* 184 3222–3241.e26. 10.1016/j.cell.2021.04.021 34004146 PMC8195859

[B125] Yu L. Jin J. Xu Y. Zhu X. (2022). Aberrant energy metabolism in Alzheimer’s disease. *J. Transl. Int. Med.* 10 197–206. 10.2478/jtim-2022-0024 36776238 PMC9901551

[B126] Zhang L. Trushin S. Christensen T. A. Bachmeier B. V. Gateno B. Schroeder A. et al. (2016). Altered brain energetics induces mitochondrial fission arrest in Alzheimer’s Disease. *Sci. Rep.* 6:18725. 10.1038/srep18725 26729583 PMC4700525

[B127] Zhang M. Li W. B. Liu Y. X. Liang C. J. Liu L. Z. Cui X. et al. (2011). High expression of GLT-1 in hippocampal CA3 and dentate gyrus subfields contributes to their inherent resistance to ischemia in rats. *Neurochem. Int.* 59 1019–1028. 10.1016/j.neuint.2011.08.023 21925558

[B128] Zhang X. X. Tian Y. Wang Z. T. Ma Y. H. Tan L. Yu J. T. (2021). The epidemiology of Alzheimer’s disease modifiable risk factors and prevention. *J. Prev. Alzheimers Dis.* 8 313–321. 10.14283/jpad.2021.15 34101789 PMC12280729

