# Supplementary figures and images for: Pre-plaque glutamatergic hyperexcitability, mitochondrial dysfunction, and dendritic remodeling in the hippocampus of one-month-old 5xFAD mice
Source: Front Aging Neurosci. 2026 Jul 15;18:1804332. doi: 10.3389/fnagi.2026.1804332 (PMC13415381; doi:10.3389/fnagi.2026.1804332)

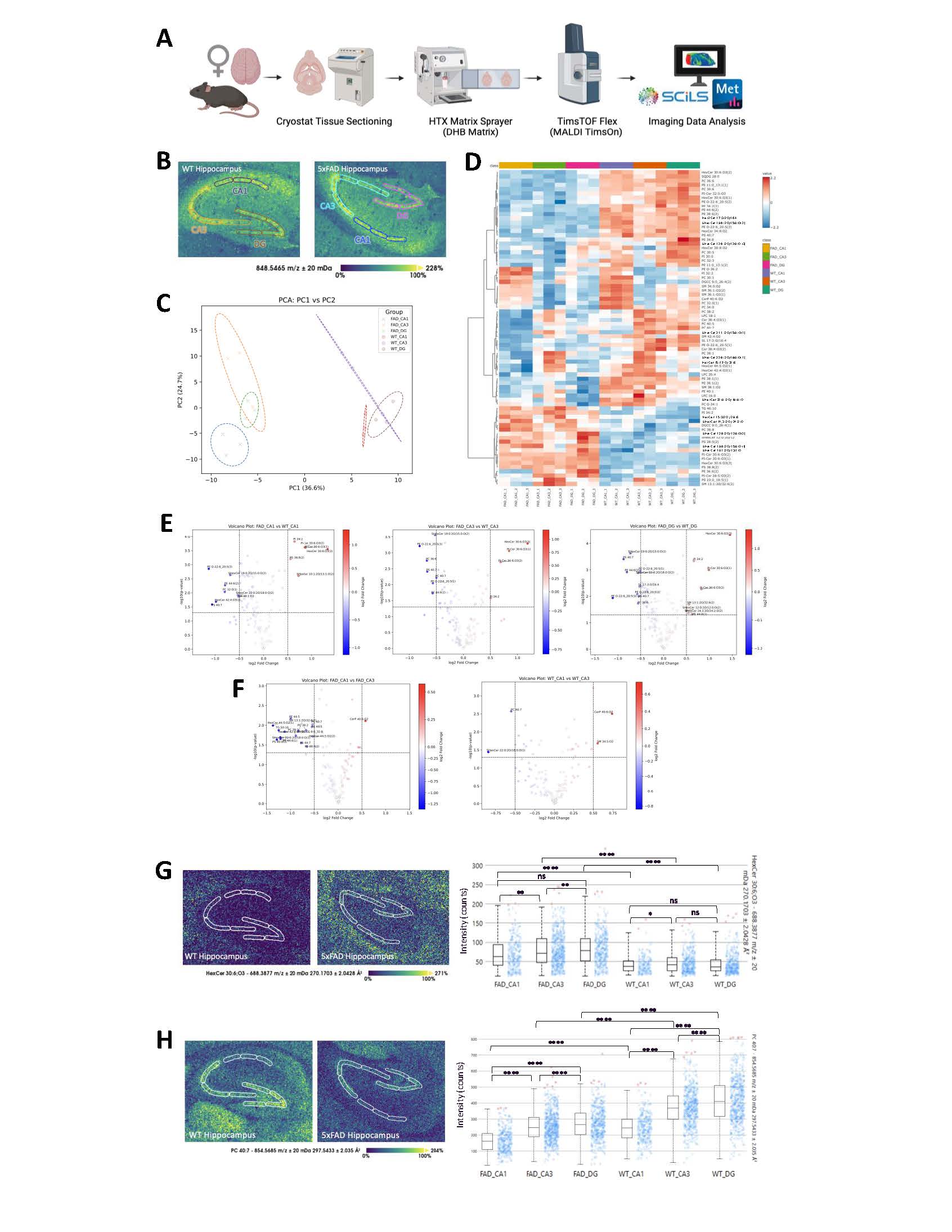

Supplement: Supplementary file 4 [file Image_3.jpeg]
